# Transcriptomic effects of paternal cocaine-seeking on the reward circuitry of male offspring

**DOI:** 10.1038/s41398-024-02839-6

**Published:** 2024-02-26

**Authors:** Nan Huang, Jian Cui, Guangyuan Fan, Tao Pan, Kunxiu Han, Kailiang Xu, Changyou Jiang, Xing Liu, Feifei Wang, Lan Ma, Qiumin Le

**Affiliations:** 1 School of Basic Medical Sciences, State Key Laboratory of Medical Neurobiology, MOE Frontiers Center for Brain Science, Institutes of Brain Science, Department of Neurology, Huashan Hospital, Fudan University, Shanghai, 200032 China; 2 Center for Biomedical Engineering, School of Information Science and Technology, Fudan University, Shanghai, 200438 China; 3 Research Unit of Addiction Memory, Chinese Academy of Medical Sciences (2021RU009), Shanghai, 200032 China

**Keywords:** Epigenetics and plasticity, Molecular neuroscience, Epigenetics and behaviour

## Abstract

It has been previously established that paternal development of a strong incentive motivation for cocaine can predispose offspring to develop high cocaine-seeking behavior, as opposed to sole exposure to the drug that results in drug resistance in offspring. However, the adaptive changes of the reward circuitry have not been fully elucidated. To infer the key nuclei and possible hub genes that determine susceptibility to addiction in offspring, rats were randomly assigned to three groups, cocaine self-administration (CSA), yoked administration (Yoke), and saline self-administration (SSA), and used to generate F1. We conducted a comprehensive transcriptomic analysis of the male F1 offspring across seven relevant brain regions, both under drug-naïve conditions and after cocaine self-administration. Pairwise differentially expressed gene analysis revealed that the orbitofrontal cortex (OFC) exhibited more pronounced transcriptomic changes in response to cocaine exposure, while the dorsal hippocampus (dHip), dorsal striatum (dStr), and ventral tegmental area (VTA) exhibited changes that were more closely associated with the paternal voluntary cocaine-seeking behavior. Consistently, these nuclei showed decreased dopamine levels, elevated neuronal activation, and elevated between-nuclei correlations, indicating dopamine-centered rewiring of the midbrain circuit in the CSA offspring. To determine if possible regulatory cascades exist that drive the expression changes, we constructed co-expression networks induced by paternal drug addiction and identified three key clusters, primarily driven by transcriptional factors such as MYT1L, POU3F4, and NEUROD6, leading to changes of genes regulating axonogenesis, synapse organization, and membrane potential, respectively. Collectively, our data highlight vulnerable neurocircuitry and novel regulatory candidates with therapeutic potential for disrupting the transgenerational inheritance of vulnerability to cocaine addiction.

## Introduction

Drug abuse is a pervasive public health crisis that has far-reaching consequences for individuals and communities worldwide. According to global estimates, approximately 275 million people between the ages of 15 and 64 have used substances at least once within the past year, among which around 13% suffer from substance use disorders (SUDs) [1]. Furthermore, studies have found that children of parents who have undergone substance abuse have a higher propensity for developing particular self-health conditions [2–5]. Previous studies have attempted to explain the causes of intergenerational inheritance, focusing mainly on the effects of maternal drug exposure during pregnancy on offspring [6–10]. However, recent evidence suggests that paternal drug use can also exert profound consequences on future generations [11, 12]. Offspring of fathers with a history of cocaine use show behavioral changes such as cocaine resistance [13], reduced sensitization to cocaine [14], impaired memory [15], and enhanced anxiety-like behaviors [16]. Previous investigations in our laboratory have provided evidence that male rats showing high seeking motivation for cocaine in cocaine self-administration (SA) behavior could transmit vulnerability to drug reinforcement to descendants, whereas yoked animals receiving the same dose of cocaine injection at the same time, were resistant to cocaine-seeking behavior [17]. The comparison of the two groups allows us to distinguish between factors of active drug seeking, leading to vulnerability to drug reinforcement, from “drug exposure” factor, leading to protective effects of cocaine resistance. However, the specific effects of these factors on the central nervous system of offspring have remained unclear.

**Fig. 1:**
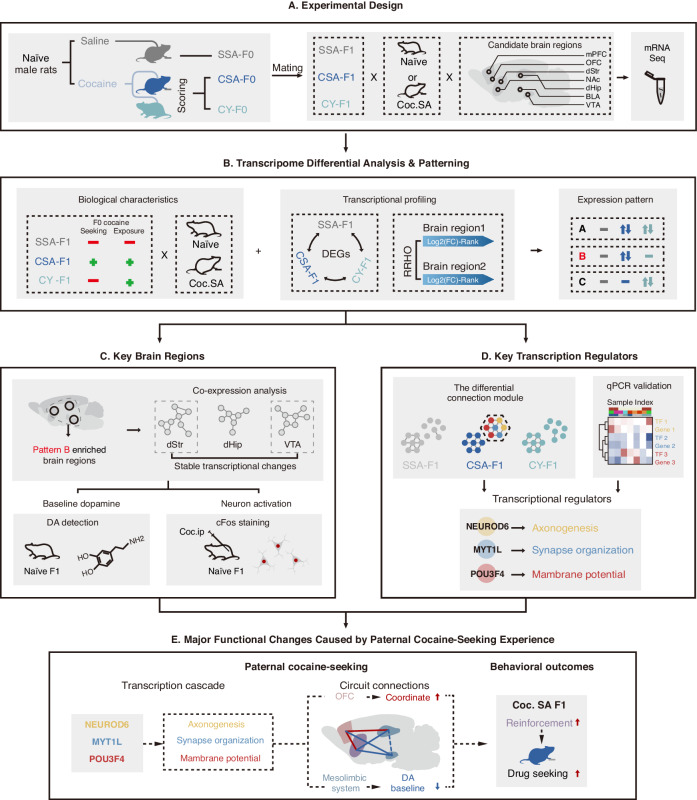
Experimental schematic for sampling and integrative analysis. **A** Experimental design. Naïve male rats were randomly assigned to perform saline self-administration (SSA), cocaine self-administration (CSA), or yoked infusions of cocaine (CY) when the contingent CSA rat received cocaine injections. The F1 generation of each group was obtained by mating with naïve female rats. F1 generation rats were subjected to cocaine self-administration tests (Coc. SA) or housed in homecage (Naïve). Candidate brain regions (OFC, mPFC, NAc, dStr, dHip, BLA, VTA) were collected and subjected to RNA-seq and data analysis. **B** Transcriptome differential analysis and patterning. Differential expression analysis across each group under naïve or Coc.SA conditions (CSA-F1 vs SSA-F1, CSA-F1 vs CY-F1, CY-F1 vs SSA-F1) were performed to evaluate the broad transcriptome changes caused by paternal- or Coc.SA-induced transcriptome changes in each brain region. RRHO across brain regions was performed to evaluate the coordinated regulation of genes across brain regions. Candidate DEGs were attributed to different expression patterns related to parental drug-seeking experience for further biological significance interpretations. Pattern B genes, possibly responding to parental cocaine-seeking but not non-contingent cocaine injections, were stressed in subsequent analyses. **C** Key brain region screening and biological verifications. Pattern B gene-enriched brain regions were selected, and the complication of all DEGs in the selected regions was subjected to co-expression analysis and functional annotation. Resting-state dopamine levels in all candidate brain regions were determined by HPLC. c-Fos^+^ cell counts after acute cocaine injection were used to assess cocaine responses in naïve F1 animals. **D** Co-expression and transcriptional regulatory mechanisms. Potential co-expression modules and key transcription regulators associated with Pattern B were identified by comparing the structure of the co-expression network across all groups. Quantitative PCR was used to verify the co-expression pattern analyzed. Co-regulatory networks by key transcription factors were deduced. **E** Major functional changes caused by paternal cocaine-seeking experience. Based on transcriptional data and biological verification, we summarized the key features of the offspring from transcriptional regulation to behavioral differences. Our analysis identified crucial transcriptional factors, including MYT1L, POU3F4, and NEUROD6, in the paternal cocaine-seeking-induced neuron-related transcriptional changes observed in the offspring. The transcriptional changes can be separated into two distinct categories: (1) “pharmacological” factor-induced evoked changes in the OFC and (2) “psychological” factor-induced changes in the limbic system. Additionally, our findings revealed that the dStr of CSA-F1 exhibits stronger reinforcement during Coc.SA, which could lead to an increased tendency towards drug-seeking behavior.

The development of susceptibility to specific drugs in offspring may arise from systemic alterations in the reward circuit during the developmental process. Various brain regions within the reward circuit play distinct roles in the drug addiction process [18, 19]. Notably, the VTA plays a crucial role as a significant source of dopamine in the brain [18], and the VTA-Str pathway has been proposed as a pivotal route contributing to cocaine addiction [20, 21]. Conversely, OFC-Str projection has been suggested to be associated with the development of compulsive drug use behaviors [19, 22]. Additionally, the hippocampus may play a role in the formation of drug dependence through its involvement in goal-directed behaviors [23, 24]. Existing studies of developmental brain disorders suggest that impairments in brain function may originate from functional impairments in hub genes, leading to widespread adaptions across the brain. Current studies on how parental drug addiction affects the central nervous system of offspring have primarily focused on individual brain regions or specific genes [14, 16, 25]. The comprehensive landscape of these changes remains undisclosed, and uncovering these alterations could provide more profound insights into the neurological consequences of cocaine addiction on offspring. Here we utilized our previous model to study the neuroadaptations caused by paternal cocaine self-administration, incorporating comparisons between cocaine self-administration (CSA), yoked cocaine infusion (CY), and saline self-administration (SSA) (Fig. 1A), and performed mRNA sequencing on seven brain regions associated with reward, under drug-free state or after (Fig. 1B). The transcriptional results were preliminarily verified by changes in assays on monoamine transmitters and cellular activation levels in response to cocaine (Fig. 1C). Through identifying various transcription factors and quantitative validation of core genes, we sought to uncover the underlying mechanisms of the transgenerational inheritance of drug-seeking (Fig. 1D). Finally, we proposed a basic model to summarize the transgenerational inheritance changes induced by paternal highly-motivated cocaine-seeking (Fig. 1E).

## Methods and Materials

Experiments were conducted in strict accordance with the National Institutes of Health Guide for the Care and Use of Laboratory Animals. Detailed descriptions of experimental design and approaches are provided in Supplemental Methods.

## Results

### Paternal highly-motivated cocaine-seeking experience enhanced cocaine self-administration in offspring

**Fig. 2:**
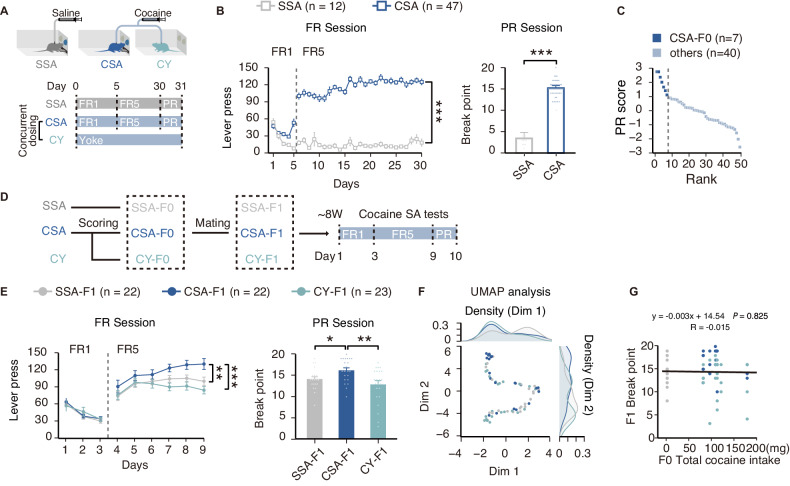
Paternal highly-motivated cocaine-seeking leads to enhanced cocaine self-administration in male F1 rats. **A** Schematic of cocaine self-administration model in F0 generation. Naïve male rats were randomly assigned to perform saline self-administration (SSA), and cocaine self-administration (CSA), or receive yoked infusions of cocaine (CY) at the same time and same dose of contingent CSA rat. Each rat in CSA and SSA groups performed self-administration for 30 days under fixed-ratio (FR) paradigm and underwent progressive-ratio (PR) test on day 31. **B** Performance of self-administration in F0 generation. The lever presses of CSA and SSA rats under the FR program (left, *P* < 0.001, Mixed model, repeated measures (MMRM)) and break point in the PR test (right, *P* < 0.001, Wilcoxon rank-sum test). SSA, *n* = 12; CSA, *n* = 47. **C** Rank of CSA individuals based on normalized Breakpoints (PR score), Top seven rats that achieved the highest score during the PR session were used for mating. **D** Schematic of offspring generation and behavior tests. Selected CSA and CY pairs, together with randomly chosen SSA rats were used to generate F1 offspring. 2-4 rats were randomly chosen from each litter to perform cocaine self-administration including 9-day FR training and PR test on day 10. **E** Performance of self-administration in the F1 generation. The lever presses of CSA-F1, CY-F1, and SSA-F1 rats in the FR (left, ***P_CSA-F1 vs CY-F1_ < 0.001, **P_CSA-F1 vs SSA-F1_ = 0.008, MMRM) and break point in the PR test (right, **P_CSA-F1 vs CY-F1_ = 0.006, *P_CSA-F1 vs SSA-F1_ = 0.011, Wilcoxon rank-sum test). SSA-F1, *n* = 22; CSA-F1, *n* = 22, CY-F1, *n* = 23. **F** Clustering of F1 individuals by UMAP based on the self-administration performance. Density plots were used to show the distribution of behavioral performance of F1 rats in different dimensions. **G** Correlation analysis between breakpoints of F1 rats and the paternal total drug intake. *P < 0.05, **P < 0.01, ***P < 0.001. Data are shown as mean ± s.e.m.

We first established the cocaine SA procedure in outbred SD rats to generate F0 generations. Rats were randomly assigned to three groups, saline self-administration group (SSA), cocaine self-administration group (CSA), and cocaine yoked-injection group (CY). SSA and CSA groups were subjected to voluntary lever-pressing for saline or cocaine for 30 days under a fixed ratio program (5-day FR1, followed by 25-day FR5), while CY was kept in identical but lever-omitted chambers, programmed to passively receive the same dose of the cocaine while the paired CSA animal received each cocaine injection (Fig. 2A). A progressive ratio test (PR) succeeded in the FR sessions and was used to evaluate motivation for cocaine seeking in CSA (Fig. 2B). To discern rats with high seeking motivation for cocaine, we scored the 47 CSA rats using the PR lever pressed, and a clear drop in score appears in the top eighth of the 47 individuals. In this way, we designated the top 7 rats (14.9%) as “highly motivated” rats (Fig. 2C), which approaches the rate (< 20%) of drug users who become addicted [1, 26]. Seven “highly motivated” rats, together with their yoked animals, and seven SSA controls, were randomly chosen and mated with naïve female rats to generate F1 (Fig. 2D). SSA-F1, CY-F1, and CSA-F1 rats (7 litters respectively) were obtained, and 3-4 adult male rats from each litter were subjected to the cocaine self-administration tests (Fig. 2D). Compared with SSA-F1 and CY-F1, the CSA-F1 rats exhibited higher lever presses for cocaine injections during the FR program and higher break points in the PR program (Fig. 2E, FR5: *P*_CSA-F1 vs CY-F1_ < 0.001, *P*_CSA-F1 vs SSA-F1_ = 0.008; PR: *P*_CSA-F1 vs CY-F1_ = 0.006, *P*_CSA-F1 vs SSA-F1_ = 0.011). At the same time, we calculated per-litter-averaged lever press to ensure that the enhanced cocaine-seeking behavior presented by CSA-F1 stemmed from inter-group differences induced by the paternal cocaine acquisition paradigm (Figure S1A, B, right). We also conducted dimension reduction analysis with behavioral characteristics, including active, and inactive lever in drug, no-drug session in FR, and PR during the self-administration. Interestingly, F1 rats’ behavioral performance diverged in Dimension 1 of UMAP according to parental drug intake, and diverged in Dimension 2 according to paternal drug-seeking experience (Fig. 2F). Moreover, correlation analysis showed that the offspring’s cocaine-seeking motivation was not correlated with paternal cocaine intake (Fig. 2G), which restated our previous findings that paternal highly-motivated drug-seeking experience, but not drug exposure per se, leads to a higher level of drug-seeking in the F1 generation.

### Paternal highly-motivated cocaine-seeking induced differentiated transcriptional responses in cortical and mesolimbic regions of F1 generation

**Fig. 3:**
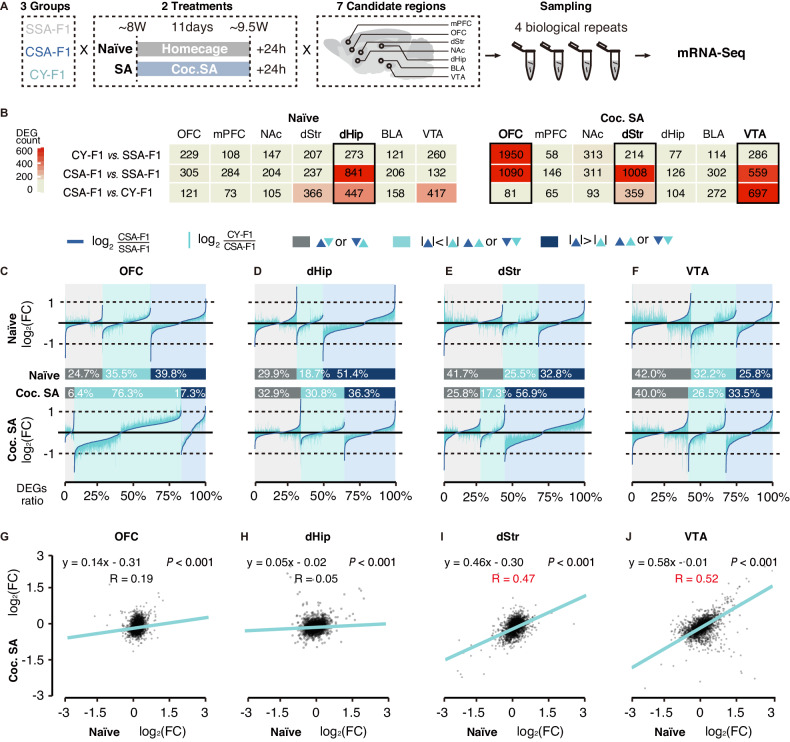
Transcriptional profiles in F1 generation are disrupted by paternal highly-motivated cocaine-seeking history throughout the reward circuitry. **A** Schematic of experimental design. Three-to-four rats from each group of SSA-F1, CSA-F1, and CY-F1 were randomly assigned to perform cocaine self-administration (Coc. SA) or remained in home cage (Naïve). Twenty-four hours after the last SA training, microdissections of seven reward-associated brain regions in 3 groups of F1 generation under Naïve and Coc. SA states were collected and subjected to mRNA-seq. **B** Table of DEG counts (CSA-F1 vs CY-F1, CSA-F1 vs SSA-F1, CY-F1 vs SSA-F1) in 7 reward-associated brain regions. Black solid frames, regions with ≥ 400 DEGs across all groupwise comparisons. **C**–**F** Summary of all DEGs by fold change consistency between CSA-F1 vs. SSA-F1 and CY-F1 vs. CSA-F1in four brain regions, OFC (**C**), dHip (**D**), dStr (**E**), and VTA (**F**). The DEGs are classified into three categories: (1) grey: CSA-F1/CY-F1 reverse changes compared to SSA-F1, (2) light blue: greater magnitude of change in CY-F1, and (3) dark blue: greater magnitude of change in CSA-F1. Within each category, DEGs are ranked based on log_2_(FC) (CSA-F1 vs. SSA-F1). The expression level of DEGs in SSA-F1 is set as references. The abscissa represents DEGs, the ordinate represents changing the magnitude of each DEG in CSA-F1 and CY-F1. The dark blue line indicates the log_2_(FC) (CSA-F1 vs. SSA-F1) and light blue vertical bars represent the log_2_(FC) (CY-F1 vs. CSA-F1). **G**–**J** Correlation of log_2_(FC) (CSA-F1 vs. CY-F1) between Naïve and Coc. SA states in OFC (**G**), dHip (**H**), dStr (**I**), and VTA (**J**).

To comprehensively analyze the potential mechanism underlying the increased drug-seeking in CSA-F1, RNA sequencing was performed in the above 3 groups, in seven brain regions including the orbitofrontal cortex (OFC), medial prefrontal cortex (mPFC), nucleus accumbens (NAc), dorsal striatum (dStr), dorsal hippocampus (dHip), amygdala (BLA), and ventral tegmental area (VTA). Two different conditions, drug-naïve (Naïve) and cocaine self-administration (Coc. SA) conditions, were included in the hope of modeling differences in the innate and cocaine abuse-induced changes in neuronal plasticity over time (Fig. 3A).

First, we used DEG counts to grossly evaluate the extent of changes in all groupwise comparisons in two states separately (Fig. 3B), as well as changes by cocaine administration in all F1 groups (Figure S3A-C). Four brain regions, including dHip, OFC, dStr, and VTA, exhibit significant between-group variance (DEG count >500) under naïve or Coc.SA-experienced states (Fig. 3B). Among these regions, OFC exhibited cocaine self-administration-magnified between-group variance in DEG number (Fig. 3B). To further differentiate if the changes in DEG number between naïve and Coc.SA states were conserved, and fold changes of CSA-F1 vs. SSA-F1 were ranked, compared with CSA-F1 vs. SSA-F1, and plotted, as shown in Fig. 3C-F. As indicated, 76.3% of DEGs showed a greater magnitude of change in CY-F1 (Fig. 3C) in response to cocaine self-administration (Figure S3B-D). While in dHip, the significant between-group variance under drug-free state diminished after Coc. SA (Figs. 3B, D). Interestingly, the transcriptional profiles of OFC and dHip in naïve and Coc. SA states were not strongly correlated (Fig. 3G, H, Figure S4A, B, E, F, I). Besides, a significantly larger number of DEGs were observed in dStr and VTA in response to Coc.SA experience (Fig. 3B), and the changes in dStr and VTA between CSA-F1 and CY-F1 were positively correlated between naïve and Coc.SA states (Fig. 3I, J, Figure S4I). In addition, the percentage of classified changes in VTA was relatively stable under both naïve and Coc. SA states (Fig. 3F). Therefore, in summary, differences across groups in cortical structures, OFC and dHip, were not maintained after cocaine self-administration (Fig. 3G, H, R _OFC_ = 0.19; R _dHip_ = 0.05) whereas subcortical mesolimbic structures, dStr and VTA, exhibited stable features (Fig. 3I, J, R _OFC_ = 0.47; R _dHip_ = 0.52), indicating that cocaine-seeking magnified the changes that already exist under naïve state in these regions.

### Pattern analysis disentangles the transcriptomic effects of paternal cocaine exposure, motivation, and passive infusion on offspring

**Fig. 4:**
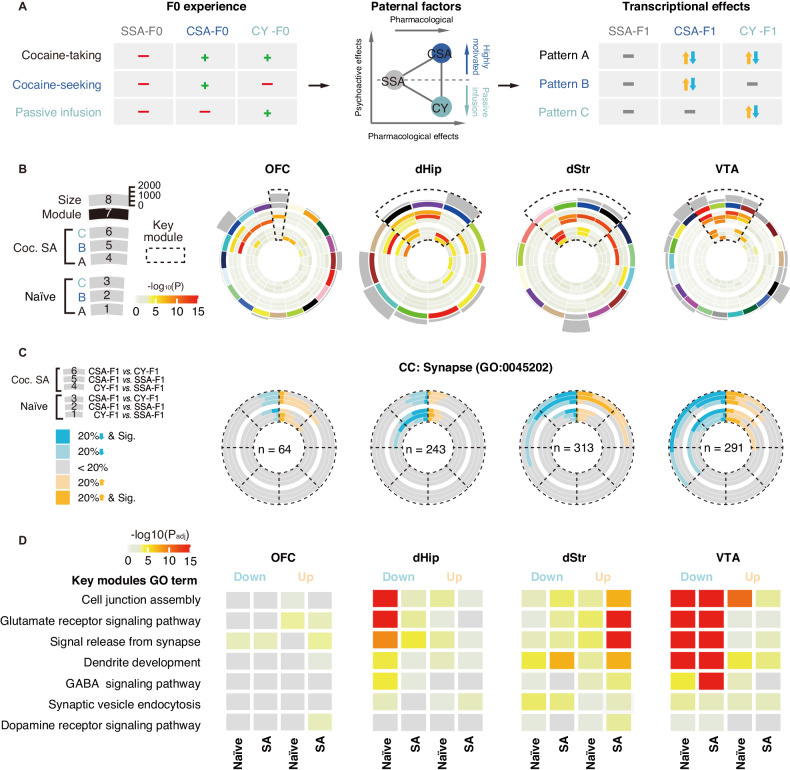
Pattern analysis reveals key brain regions and pathway changes attributable to paternal highly motivated cocaine seeking. **A** Schematic of sorting method for categorizing genes in pattern analysis. F0 rats undergo distinct cocaine-acquired processes. CSA-F1 is uniquely affected by paternal highly-motivated cocaine-seeking, CY-F1 is uniquely affected by paternal passive cocaine infusion, and CSA-F1, and CY-F1 are unified affected by paternal cocaine exposure. Three expression patterns that represented the “pharmacological”, “highly-motivated”, and “passive infusion” impact of the paternal behavioral paradigm were picked out. **B** Circos plots for the WGCNA result of OFC, dHip, dStr, and VTA. Each slice in the chart represents a gene co-expression module, with the outermost representing the modular size of the module. The secondary outer rectangle indicates an arbitrary color used to name the module. The inner concentric circles represent the degree of enrichment for pattern genes, with colors reflecting Fisher’s exact test (FET) *P* values. Dashed lines, and modules enriched with pattern B. **C** Pie charts of group differences of OFC, dHip, dStr, and VTA in synaptic-enriched genes within the screened module before and after cocaine self-administration (blue = significantly down (20% downregulation, *P* ≤ 0.05), light blue = not significantly down (20% downregulation, *P* > 0.05), yellow = significantly up (20% upregulation, *P* ≤ 0.05), light yellow = not significantly up (20% upregulation, *P* > 0.05). **D** Enrichment analysis (CSA-F1 vs. CY-F1) for the representative subset of synaptic function in OFC, dHip, dStr, and VTA (colors reflect adjusted *P-*values).

A notable distinction between the three F1 groups is that while both CSA-F0 and CY-F0 were exposed to cocaine, only CSA-F0 had voluntary access to cocaine (Fig. 4A). Although both groups received the same dose of cocaine, it is important to consider that cocaine, being a psychoactive drug [27, 28], may induce different psychoactive effects in the two groups due to the absence of operant behavior in CY rats [29]. The act of drug-taking involves the immediate ingestion or use of the drug to experience its effects, and both CSA and CY rats undergo this process. However, the crucial difference lies in the motivation behind drug-seeking. Additionally, the passive acquisition of drugs by CY rats may generate potential stress, that may obstruct drug-seeking behavior in offspring. Therefore, three key factors contribute to the paternal influence on offspring: 1) the direct effects of the drug itself, 2) the drug craving induced by voluntary drug-seeking, and 3) the potential stress induced by passive acquisition (Fig. 4A), which could be further attributed to 3 clusters according to its biological relevance: Pattern A, expression profiles as responsive to “drug exposure” factors (cocaine exposure-induced changes, i.e., consistent significant changes in CSA-F1 vs. SSA-F1 and CY-F1 vs. SSA-F1); Pattern B, genes attributed to the “highly motivated” category (Significant in CSA-F1 vs. SSA-F1 & CY-F1, but not changed in CY-F1 vs. SSA-F1); Pattern C genes were associated with the “passive infusion” impact (Significant in CY-F1 vs. SSA-F1 & CSA-F1, but not changed in CSA-F1 vs. SSA-F1) (Fig. 4A, S5). We unsupervisedly classified DEGs into 12 expression patterns to disentangle the effects of the three factors. The DEG counts and GO enrichment analysis within each pattern reveal that distinct paternal factors lead to specific transcriptional changes in the F1 generation (Figure S5, S6). Notably, genes related to posttranscriptional gene silencing and protein transport were uniquely influenced by Pattern A, the paternal “pharmacological” factor, in OFC, with no significant enrichment observed in Pattern B and C clusters. Conversely, dStr, dHip, and VTA were primarily affected by Pattern B, relating to the “highly motivated” factor (Figure S5, S6). Overall, the paternal psychoactive effects differ from the pharmacological effects, which exert a wide-ranging influence on multiple brain regions, particularly the mesolimbic system. These transcriptomic changes are likely to have significant implications for nervous system development and neuronal function (Figure S6).

To capture the full breadth of information in the transcriptome data and avoid overlooking key details through simple gene classification, we employed gene-weighted gene co-expression network analysis (WGCNA) [30] to summarize gene expression modules within each brain region. The impact of paternal factors on each gene module was assessed based on the enrichment degree of pattern genes (Fig. 4B). To thoroughly investigate the impact of paternal psychoactive influences on the neurological function of offspring, we specifically directed our attention to the genes annotated to the “Synapse” GO term within the enriched modules associated with pattern B and pattern C (Fig. 4C). The significance of genes, as well as annotated GO terms were used to further characterize the Pattern B changes. Notably, these changes were observed to be predominantly up-regulated in dStr, while exhibiting a down-regulated pattern in the dorsal hippocampus dHip and VTA, and no significant changes in OFC (Fig. 4C). Specifically, the synaptic differences in the dHip were exclusively observed in the drug-naïve state (Fig. 4C, Figure S7E). Conversely, the differences in synapses within the dStr and OFC exhibited more pronounced disparities between groups after cocaine self-administration (Fig. 4C, Figure S7D, F). In contrast, the ratio of groupwise differences in the VTA remained consistent across conditions (Fig. 4C, Figure S7G). As the synaptic differences observed in VTA were independent of cocaine exposure in the F1 generation, and the close neural connections between the striatum and VTA, it is plausible that the paternal psychoactive effects on the VTA may exhibit stability and have long-lasting impacts (Fig. 4C, D).

### Paternal highly-motivated cocaine-seeking reduced basal dopamine level and increased neural activation induced by cocaine in F1 rats

**Fig. 5:**
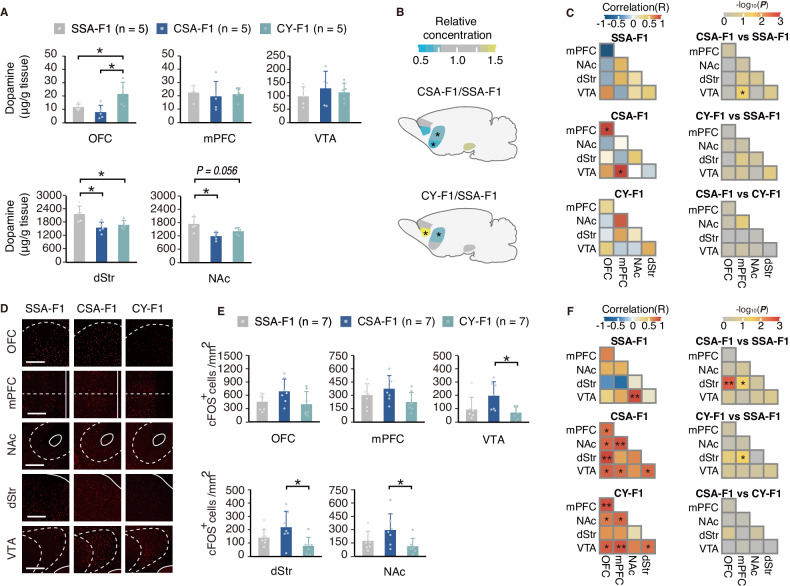
Effects of paternal cocaine addiction on dopamine baseline and cocaine-induced neuronal activation in F1 generation. **A**, **B** HPLC-based quantification of baseline dopamine level in F1 generation. **A** The concentration of dopamine in VTA and its downstream projecting regions in naïve F1 rats (*P_OFC: CSA-F1 vs CY-F1_ = 0.032, *P_OFC: CY-F1 vs SSA-F1_ = 0.032, *P_NAc: CSA-F1 vs SSA-F1_ = 0.016, *P_dStr: CSA-F1 vs SSA-F1_ = 0.032, *P_dStr: CY-F1 vs SSA-F1_ = 0.032, Wilcoxon rank-sum test, *n* = 5 per group). **B** Heatmap shows the relative changes in concentration of CSA-F1 and CY-F1 compared to SSA-F1. **C** Correlation analysis of the dopamine level across brain regions. Left, the degree of correlation (R) between two brain regions, Pearson correlation test. Right, the significance of the correlation (*P*) of dopamine concentrations between brain regions across each group. **D**, **E** Cocaine-induced neuronal activation by c-Fos staining in VTA and its major downstream 1 hr after acute cocaine injections (i.p., 10 mg/kg) in F1 generation. **D** Representative immunofluorescence staining. Scale bar, 100 μm. **E** Statistics of c-Fos^+^ cell density (*P_NAc: CSA-F1 vs CY-F1_ = 0.026, *P_dStr: CSA-F1 vs CY-F1_ = 0.038, *P_VTA: CSA-F1 vs CY-F1_ = 0.026, Wilcoxon rank-sum test, *n* = 7 per group). **F** Correlation analysis of the density of c-Fos^+^ cells in VTA and its major downstream. Left, the degree of correlation (R) between two brain regions, Pearson correlation test. Right, the significance of the correlation (*P*) of c-Fos^+^ cell density between brain regions across each group. *P < 0.05, **P < 0.01. Data are shown as mean ± s.e.m.

Dopamine-releasing neurons located in the ventral tegmental area (VTA) play crucial roles in reward-related and goal-directed behaviors [18, 19, 22]. Given the central role of the VTA in transcriptomic analysis, we hypothesized that dopamine-centered rewiring of the midbrain circuit could increase cocaine seeking of the CSA offspring. HPLC-based monoamine neurotransmitter quantification of naïve F1-generation rats (*n* = 5 per group) revealed reduced dopamine and dopamine metabolites content in the OFC, NAc, and dStr of CSA-F1 (Fig. 5A, B, Figure S8A, B). Furthermore, mPFC DOPA level exhibited a significant positive correlation with OFC and VTA in CSA-F1 (Fig. 5C). The decrease in dopamine levels and the altered correlation of basal dopamine content between nuclei suggest that VTA plays a key role in the regulation of the high drug-seeking motivation phenotype in the CSA-F1 group, which is in general agreement with the transcriptomic results.

Decreased baseline dopamine level may lead to D2 disinhibition and potentially result in increased cellular activation. To assess cocaine-induced neuronal activation, 10 mg/kg of cocaine was intraperitoneally injected into each naïve F1 rats and then sampled after 1 hr. cFos^+^ cell density within each region was calculated (Fig. 5D, E). Differential cFos^+^ cell density was observed in NAc, dStr, and VTA (Wilcoxon rank-sum test, NAc, *P*_CSA vs CY_ = 0.026; dStr, *P*_CSA vs CY_ = 0.038; VTA, *P*_CSA vs CY_ = 0.026). The results showed that highly-motivated paternal cocaine-seeking caused enhanced activation in the offspring upon cocaine exposure (Fig. 5E). Paternal cocaine exposure in CSA-F1 and CY-F1 has been found to potentially induce a heightened co-activation pattern in the reward circuitry of offspring (Fig. 5F, left), and in particular, OFC and VTA correlated more strongly with other nuclei. This result may correlate with the down-regulation of dopamine levels and also suggests that there may be coherent changes in the way nuclei correspond to cocaine, prompting us to explore the possible role of coherent changes in inter-nucleus transcripts on drug-seeking behavior in CSA-F1.

### Modular transcription factor regulation underlies the coordinated transcriptional changes across brain regions of F1 generation

**Fig. 6:**
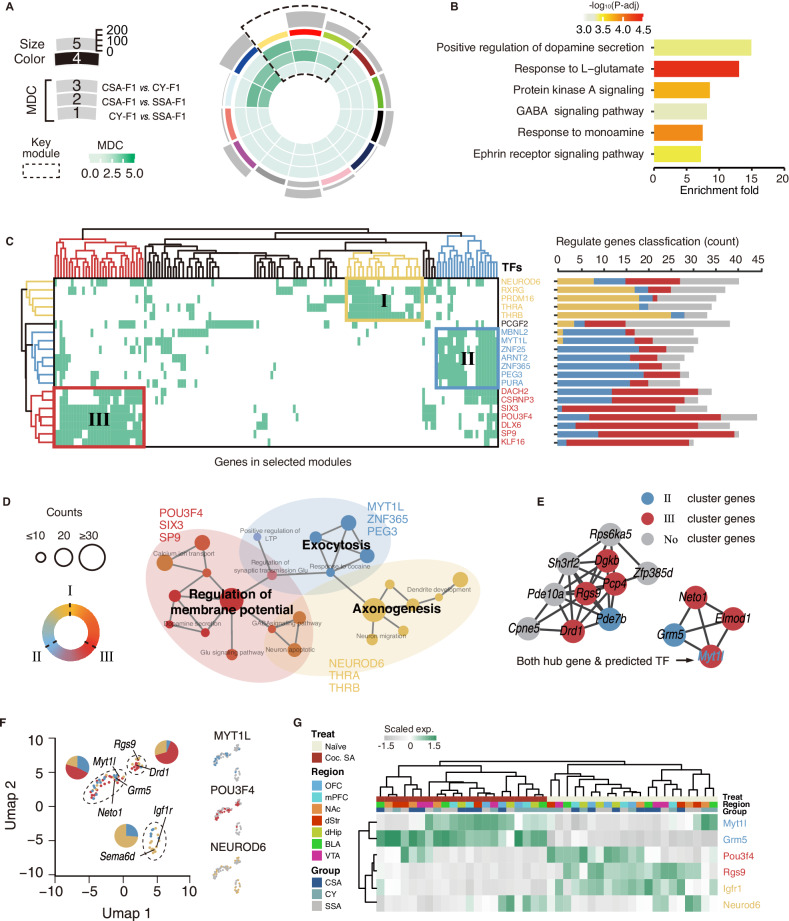
The presumed gene co-regulatory network within the offspring’s reward-associated brain regions. **A** Circos plots for the WGCNA result of all pattern genes across seven brain regions in CSA-F1. Each slice of the chart represents a gene co-expression module. The outermost represents the modular size of this slice. The secondary outer rectangle is an arbitrary color as the module name. The innermost concentric circles represent the modular connective difference (MDC) between networks provided in Figure S10A, B. Modules selected for subsequent analysis are marked with dashed lines. **B** Pathway analysis of screened modules in **A**, colors reflect adjusted *P* value in enrichment analysis. **C** The regulatory effects of predicted transcription factors on modular genes. Each row reflects one of the top 20 predicted transcription factors, each column reflects a gene in dashed modules in **A**. The grid is marked in color and numbered if the gene is predicted to be regulated by the transcription factor (left). Bar charts show the percentage of genes within each regulatory cluster (right). **D** Enriched pathways of each transcription regulatory cluster. Edge represents the gene overlap across GO terms. Node size represents the gene counts in each GO term. Color represents the fraction of genes in each cluster. A light-colored shading denoted the GO terms which are controlled by major transcriptional factors. **E** Screening hub genes of predicted differential regulatory modules in **A**. The top 15 are displayed using the network diagram. The color of nodes represents the cluster to which the node belongs as noted in **C**. grey = no cluster, blue = Cluster II, red = Cluster III. **F** Dimensionality reduction analysis of candidate genes based on whole-brain gene expression. Colors represent clusters of transcriptional regulation predicted based on transcription factors, red = cluster I, blue = cluster II, red = cluster III. **G** Independent qPCR validation was performed on the predicted transcription factors and their downstream, and detailed results are provided in Figure S12. Colors represent Scaled expressions. The index of the sample is provided on top of the heatmap.

To evaluate the potential synergy of transcriptomic changes in different reward-associated brain regions, we ranked genes in each region based on their *P*-values (CSA-F1 vs SSA-F1 and CY-F1 vs SSA-F1) and then utilized rank-rank hypergeometric overlap (RRHO2) [31, 32] to assess the similarity of gene ranking between different regions (Figure S9). In the naïve state of CSA-F1, there was a more coordinated up- and downregulation of transcriptional signatures between the OFC and mPFC, vs. dStr, dHip, and VTA, as revealed by RRHO analysis (maximum *Fisher*’s exact test (FET) *P* < 1.0 × 10^−50^) (Figure S9 upper-left). An interesting switch exists in dStr vs. mPFC, and OFC vs. mPFC on Coc. SA, as all genes showed a high degree of inversion coordination. To gain deeper insights into the molecular mechanisms underlying the transgenerational inherent vulnerability to cocaine reinforcement across multiple brain regions, we performed multi-brain-region co-expression network analyses in SSA-F1, CSA-F1, and CY-F1 rats. This approach allowed us to integrate data from all seven brain regions studied, utilizing an established pattern gene set (union of A, B, and C pattern genes in Figure S5). We next used module differential connectivity (MDC) analysis (detail provided in Supplementary Materials) to quantify changes in network connectivity between groups (Fig. 6A for CSA-F1, Figure S10A for SSA-F1, S10B for CY-F1). We screened the modules that were potentially differentially regulated in CSA-F1 based on MDC (Fig. 6A, black dashed lines). The enrichment of the GO term supports the hypothesis that neuroplastic changes in reward circuitry affected by paternal highly motivated cocaine-seeking induce vulnerability to cocaine reinforcement in offspring. (Fig. 6B). By analyzing the conservation of the gene modules, the screened modules were remodeled by the parental cocaine-seeking experience, suggesting a potential transcriptional regulatory mechanism (Figure S10C).

To find the key nodes in the transcriptional regulation process, we performed transcription factor prediction analysis using ChEA3 [33]. Based on the expression profiles, predicted candidate transcription factors were clustered into three groups (Fig. 6C). Cluster I is primarily regulated by NEUROD6, THRA, and THRB, Cluster II by MYT1L, ZNF25, and PEG3, and Cluster III by SIX3, POU3F4, and SP9, etc. Some genes exhibited patterns under co-regulation of Cluster II and III transcriptional factors, such as genes annotated under DACH2 and CSRNP3 (Fig. 6C). When assigned to brain regions, genes in Clusters I and III were found to be predominantly expressed in VTA and dStr, while Cluster II did not show a specific regional distribution pattern (Figure S10D). Additionally, the main signaling pathways involved in each cluster and downstream of transcription factor were distinct (Fig. 6D). For example, genes in Cluster I are involved in neuron migration and dendrite development regulated by NEUROD6, genes in Cluster II are involved in exocytosis and response to cocaine regulated by MYT1L, and genes in Cluster III are involved in neurotransmitter signaling and regulation of membrane potential regulated by POU3F4 (Fig. 6D). Next, the top 15 genes with the most molecular interactions in the co-expression network were chosen as hub genes to generate a co-expression network (Fig. 6E). The final network revealed that genes in Cluster II and III act as the hub nodes. Notably, MYT11L was identified as a key transcriptional regulator and hub of the gene network, leading to the hypothesis that it plays a crucial role in the transmission of vulnerability to cocaine across generations (Fig. 6E). To confirm the validity of the bioinformatic analysis relying on transcriptional network alterations and potential transcription factors, we conducted unsupervised dimensionality reduction on the global brain-scale expression of all candidate genes. This analysis revealed that the expression patterns of the candidate genes across the entire brain were similarly grouped into three categories, aligning with the outcomes of the transcription factor prediction (Fig. 6F). Furthermore, we carried out independent qPCR validation for two key candidate genes within each group, and the results consistently matched the expression patterns observed in the sequenced samples (Fig. 6G). These results underscore the reliability of our bioinformatics analysis and further revealed that Cluster II and III mainly represent differential Coc. SA-induced changes, while Cluster I, with main GO annotation of axonogenesis, neuronal migration, and dendrite development, likely drive the developmental diversity between the three groups.

In conclusion, transcriptional hubs that underly shared transcriptional changes across multiple reward-associated brain regions were identified in CSA-F1 rats, and these hubs may lead to the specific transcriptomic profile of reward circuitry in CSA-F1 rats. These findings plan a general direction for the subsequent exploration of the transgenerational effects of paternal highly-motivated drug-seeking.

## Discussion

Prior studies have primarily concentrated on parental-induced germ cell changes during intergenerational inheritance, yielding significant findings related to DNA methylation, modifications in histones, and noncoding RNAs in germ cells [3, 34, 35]. However, there has been limited exploration into the broader impact of these epigenetic changes on the central nervous system (CNS) of adult offspring [25]. Additionally, existing histological analyses have often been confined to single-brain region perspectives, such as research focused on transcriptomic alterations in the offspring’s hippocampus influenced by maternal glucocorticoid exposure during pregnancy [36] and the transcriptomic effects of paternal adolescent stress on the offspring’s amygdala [37]. While these studies have suggested potential target brain regions, such as mPFC, NAc, hippocampus, and amygdala, their findings have yet to be comprehensively integrated. Consequently, in our study, we have investigated transcriptomic changes in seven brain regions within the reward circuit of offspring, under the influence of paternal cocaine addiction, utilizing cocaine self-administration versus yoked administration as an animal model. This research aims to provide an extensive overview of the transcriptomic effects of paternal cocaine addiction on the reward circuit of offspring (Fig. 3B, Figure S3A-C). We leveraged two statistical approaches (pattern identification and gene co-expression network analysis) to characterize gene expression patterns (Figure S5, Fig. 4A, Fig. 6A, Figure S10A, B). At the individual gene level, our findings align with existing studies, mirroring changes in BDNF expression in the forebrain region, which were both detected and identified as candidate targets [13] (Figure S10D). Moreover, our data also revealed shifts in mRNA expression of molecules related to neurogenesis, such as Btg3 and Nr4a1, consistent with prior research [17]. Taking a broader perspective encompassing multiple brain regions, we systematically delved into two key areas of investigation. First, we explored the potential mechanisms responsible for the divergent behavioral outcomes arising from simple paternal drug intake versus highly motivated drug-seeking in offspring (Fig. 4). Second, we examined potential transcriptional regulatory mechanisms driving alterations in the gene expression network within the offspring’s reward circuit (Fig. 6).

### Distinct transcriptional changes in F1 generation underly paternal pharmacological, highly motivated, and passive infusion factors

Our study has yielded significant insights into the distribution of pattern genes across seven distinct brain regions (Figure S5). Specifically, we observed a clear enrichment of paternal “pharmacological” factor-induced Pattern A genes (Figure S5). The transcriptomic effects of paternal cocaine exposure were concentrated in the OFC and triggered by recurring cocaine-seeking behavior in the offspring (Fig. 3C, Figure S4A-E, Figure S5). The effects of paternal highly-motivated seeking on the transcriptome, i.e., “highly motivated” factor, appeared to be more complex (Figure S5, which led to widespread changes in the expression of genes associated with various aspects of neural function in the offspring (Figure S6). Although the changes induced by paternal “passive infusion” were relatively minimal in terms of quantity, they still resulted in noteworthy alterations in the offspring’s transcriptome, particularly affecting processes such as neuron and glial differentiation in NAc and VTA (Figure S6). To gain a deeper understanding, we integrated transcriptome analysis with assays measuring resting-state dopamine levels (Fig. 5A-C, Figure S8) and cellular activation (Fig. 5D-F). Based on our analysis of available data and experimental results, we ventured to propose a conjecture regarding the potential mechanisms through which paternal highly-motivated cocaine-seeking may impact the reward circuitry in offspring. We suggest that these effects occur through two distinct mechanisms - (*a*) Paternal “pharmacological” effects: This mechanism involves evoked changes in the OFC. The primary function of this change is to identify substances associated with paternal exposure. - (*b*) Paternal “psychoactive effects” factor-induced changes in the mesolimbic system: This mechanism mainly induces differentiated preferences in the offspring.

Based on the distribution of pattern A genes (Figure S5, Figure S7A-C) and the stronger co-activation observed in the reward circuitry of CSA-F1 and CY-F1 (Fig. 5D, F), it appears that the paternal “pharmacological” effects may have a significant impact on the formation of connections in OFC (Figure S5, Figure S6, Figure S7A-C). The OFC plays a crucial role in integrating new information with pre-existing cognitive frameworks and associating values with specific events [24, 38]. Therefore, the distinct network patterns observed in the OFC of the F1 generation could potentially mediate changes in how offspring respond to substances related to paternal exposure. Even if this idea is a simple conjecture at the moment, the fact that cocaine exposure does not affect the offspring’s sucrose-seeking or nicotine-seeking behavior seems plausible [13, 14, 17, 39].

In contrast, transcriptomic changes in the mesolimbic system induced by paternal psychoactive effects appeared different. VTA shows stable down-biased transcriptomic change and dHip and dStr exhibit relative labile transcriptomic changes (Fig. 4C, Figure S4I). In our results, the plasticity changes in the VTA-striatum circuits in the transgenerational inheritance of vulnerability to cocaine were comprehensive. The lower dopamine baseline in the offspring, which is consistent with previous studies in highly addicted rats in neurochemistry [40], (Fig. 5A, B) may be linked to the stable transcriptomic changes occurring in the VTA. This alteration in dopamine baseline could have a significant impact on individual decision preferences and may be critical in accounting for individual differences in drug susceptibility [41–44]. However, it is important to note that changes in dopamine baseline alone cannot fully explain the behavioral changes in offspring, as the alterations in dopamine baseline in response to both “highly motivated” and “passive infusion” factors appear similar in the striatum (Fig. 5A, Figure S8C). Stronger response to cocaine (Fig. 5E) may be a key factor leading to changes in drug susceptibility in offspring [40, 45]. We also observed transcriptomic changes in the dHip induced by the “highly motivated” factor (Fig. 4B), up-regulation of the glutamate receptor signaling pathway in the dStr (Fig. 4D), and co-activation changes in response to cocaine exposure (Fig. 5D-F). Integrating the above evidence we speculate that the difference in dopamine baseline with distinct responding patterns to cocaine (Fig. 5) affects the long-term potential of striatal spiny projection neurons (SPNs). In addition, OFC-dStr circuits, which establish compulsive behavior [46] and present a stronger coactivity in CSA-F1 (Fig. 5F), may also participate in the distinct striatal plasticity in F1 generation. As a result, the differences in reinforcement to drug may result in the stabilization of innate differences and mediate the distinct vulnerability to cocaine in the F1 generation. However, it is essential to acknowledge that this paper does not have enough evidence to fully support this argument; it merely proposes this hypothesis as a potential avenue for further investigation and understanding of the underlying mechanisms.

### Potential regulatory mechanisms of intergenerational inheritance of cocaine addiction

Previous research has indicated that certain transcription factors confer risk for drug addiction [47–49]. Additionally, drug addiction would reshape transcriptome-wide responses in the central nervous system, a process likely mediated by specific transcriptional regulatory mechanisms [48, 50–52]. In light of these insights, we constructed a gene expression network by amalgamating transcriptomic data from various brain regions both before and after cocaine administration. Our objective was to examine alterations in this gene expression network to gain insights into the intergenerational genetic mechanisms of drug addiction and their downstream regulatory effects. We identified key transcription factors, such as MYT1L, POU3F4, NEUROD6, etc. (Fig. 6C), which are involved in nervous system development [53–56] but have diverse downstream functions (Fig. 6D). MYT1L is widely distributed in the brain, while POU3F4 and NEUROD6 have specific spatial and temporal distributions [57]. POU3F4 plays a role in early neurodevelopment and regulates membrane potential-related genes (Fig. 6D), while NEUROD6 is concentrated in the cerebral cortex and regulates axonogenesis-related genes (Fig. 6D). Besides, MYT1L is a potential downstream of POU3F4 [58, 59]. We speculate that POU3F4 mediates epigenetic signals that induce neuronal precursor differentiation during embryonic development and cause a transcriptional cascade [60–62] of transcription factors and genes related to membrane potential (Fig. 6D). This may explain why some genes have similar expression patterns across the brain but show different expression changes between brain regions (Figure S11).

In conclusion, our study provides a unique transcriptomic resource of the reward circuits in the context of paternal cocaine addiction. Through large-scale transcriptomic analysis, we gained an initial understanding of the molecular basis of intergenerational genetic effects of highly-motivated drug-seeking across multiple brain regions. This work partially explains the intergenerational genetic mechanisms of vulnerability to cocaine and suggests potential targets for reversing the negative intergenerational effects. However, we must exercise caution in generalizing our results from male rats to females, as even the most basic behavioral phenotypes differ significantly between the sexes [13, 16]. It is important to emphasize that this paper introduces a novel multi-brain transcriptome perspective on the intergenerational genetic effects of cocaine, providing new insights into this intricate process. Nevertheless, we acknowledge that these findings are exploratory and warrant further in-depth research and validation.

### Supplementary information

Supplement information

Statistical Analysis

Key Resources Table

## Data Availability

All data are available in the main text or the supplementary materials. Additional data related to this paper may be requested from the authors. Raw NGS data are deposited in the NCBI BioProject database under accession number PRJNA788009.
