# Supplementary material for: Transcriptomic effects of paternal cocaine-seeking on the reward circuitry of male offspring
Source: Transl Psychiatry. 2024 Feb 26;14:120. doi: 10.1038/s41398-024-02839-6 (PMC10897445; doi:10.1038/s41398-024-02839-6)
Supplement: Supplementary file 1 — Supplement information [file 41398_2024_2839_MOESM1_ESM.pdf]

Supplementary Materials for

Transcriptomic Effects of Paternal Cocaine-seeking on the Reward Circuitry of Male Offspring

Nan Huang[1†], Jian Cui[1†], Guangyuan Fan[1], Tao Pan[1], Kunxiu Han[1], Kailiang Xu[3], Changyou Jiang[1,2], Xing Liu[1,2], Feifei Wang[1,2], Lan Ma[1,2*], Qiumin Le[1,2*]

Affiliations:

[1]School of Basic Medical Sciences, State Key Laboratory of Medical Neurobiology, MOE Frontiers Center for Brain Science, Institutes of Brain Science, Department of Neurology, Huashan Hospital, Fudan University, Shanghai 200032, China

[2]Research Unit of Addiction Memory, Chinese Academy of Medical Sciences (2021RU009), Shanghai 200032, China

[3]Center for Biomedical Engineering, School of Information Science and Technology, Fudan University, Shanghai 200438, China

[†]Theses authors contributed equally to this work.

[*]Corresponding authors. Emails: lanma@fudan.edu.cn or qiuminle@fudan.edu.cn

This PDF file includes:

Materials and Methods

Figures S1 to S12

Tables S1 to S4

Materials and methods

Animal experiments

Animal

All experimental animals used in the experiments were Sprague-Dawley. Rats were maintained at 22±2°C with 40±10% humidity on a 12 h light/dark reverse cycle (lights on from 16:00) with ad libitum access to food and water. All rats were habituated to the animal facility for 1 week before experimental manipulations. Experiments were conducted strictly with the National Institutes of Health Guide for the Care and Use of Laboratory Animals and approved by the Animal Care and Use Committee of Shanghai Medical College of Fudan University.

Cocaine self-administration

Random sampling: All animals used for this experiment were uniquely numbered. During random selection of animals, the selected animal codes were generated and renumbered using the sample() function in R (e.g., original #1021 -> experimental #01) and added to the index list. The process of sampling and renumbering was done by independent experimenters not involved in subsequent experiments, and the original grouping of each rat was revealed upon completion of the experiment.

Food training: Before food training, rats require food restriction to maintain body weight at 85% of normal body weight. During the food training process, the trained rats were provided with both effective and ineffective levers. Rats were provided food pellets (BioServ, Flemington, NJ, USA) if rats press the active lever, but could not get food pellets if they pressed the inactive lever. Rats will perform the subsequent

operation, If the rats can successfully obtain 150 food pellets.

Intravenous catheterization in rats: Animals were anesthetized with isoflurane. A silicone catheter (ID = 0.31 mm, OD = 0.64 mm, Dow Corning) was implanted from the back of the rat, and the catheter was subcutaneously implanted into the right jugular vein of the rat through the neck at a depth of 3 cm. Rats were maintained for 7 days after completing the operation. During the maintenance period, 0.1 ml of normal saline containing heparin (30 IU/ml) and gentamicin (0.5 mg/ml) was used to flush the catheter. Rats with good postoperative recovery will be used for subsequent self-administration experiments.

Fixed ratio administration (FR): During the experiment, the rats received 2.5h fixed-ratio administration which included three 40-min administration periods and two 15-min non-administration periods. Retractable active and inactive levers, an ambient light, a blue cue light, and a white cue light are installed in the self-administration box. The blue clue light is on during the dosing phase, the blue clue light turns off and the ambient light turns on during the no-dosing phase. At the beginning of fixed ratio administration, the active and inactive levers stretch out and the blue cue lights turn on. If the rat presses the active lever for a prescribed number (1 time in FR1, 5 times in FR5), rats receive cocaine saline solution (750 μg/(kg*Inf)), at the same time, the blue clue light will be off, the white ambient light will be on, and the white noise clue will sound. In the non-administration stage, after the press counts of active levers reached the prescribed number, rats can't receive cocaine saline solution.

Progressive ratio administration (PR): The progressive ratio administration test was

used to assess the reinforcement motivation of rats. During the progressive pedal administration, the number of lever presses required for the rat to obtain a single dose of cocaine solution will gradually increase (i^{th} injection=Int ($5e^{0.25i-5}$)). The test was carried out for 2.5 hours. All CSA rats would be scored after the progressive ratio administration test, scoring rule: $S_i = \frac{X_i - \bar{X}}{s.d.}$, $X_i$is the active lever press during PR test for each rat, $\bar{X}$ is the mean active lever press during PR for all the rats, and s.d. is the s.d. of the population active lever press during PR. 24 hours after the last self-administration session, sorted "highly motivated" male rats were housed with two naive female rats to generate F1.

Yoked drug infusion procedure: Rats were randomly divided into the cocaine self-administration group and the yoke group. yoke rats were paired with a self-administered rat. After the self-administered rats completed the prescribed number of lever presses, the self-administered rats and the yoke rats received the same dose of cocaine saline solution intravenously. During the experiment, the blue cue light was always on in the yoke group rats, and touching the levers during the experiment did not induce cues and administration events.

Immunofluorescence staining

The rats were perfused with PBS first, then with 4% paraformaldehyde in PBS (pH 7.5), and the brains were removed. After post-fixation in 4% paraformaldehyde for 12 h, the samples were dehydrated in 30% sucrose/PBS twice. brains freeze at -80°C in optimal cutting temperature compound (Leica) and then sectioned coronally (30 μm). frozen sectioned brain tissue (30 μm) was placed in a 24-well cell culture dish, washed

with PBS solution for 10 min, and then blocked with a blocking buffer (PBS + 5% normal donkey serum, 0.5% Triton X-100) for 2 h at room temperature. then incubated with primary antibodies (c-FOS, Synaptic Systems (226008), 1:2000) overnight at 4 °C. Slices were then washed three times for 10 min each with PBS, incubated with secondary antibodies (Donkey anti-Rabbit CY3 1:1000 (Jackson Immuno Research, (715-546-151)) for 2 h, and washed three times for 10 min each with PBS, then counterstaining with DAPI and mounted on microscope slides for confocal imaging. All immunofluorescence images were imaged with the Olympus VS120 digital sectioning workstation (CY3, Ex.540/25 nm; Em.605/55 nm). ImageJ was used for subsequent image analysis, the final number of c-Fos^{+} cells was confirmed by manual counting.

Monoamine transmitter extraction

The rats were perfused with ice-cold PBS, and the brain tissue was quickly taken out on the ice and weighed in a 1.5 ml centrifuge tube. Add 10 times the volume of 4% perchloric acid solution, then homogenize and stand the homogenized brain tissue on ice for 30 min. After standing, centrifuge at 9600 × g for 15 min at 4°C to take out the supernatant, repeat twice to ensure that there is no impurity in the supernatant, and store it in a -20°C refrigerator for high-performance liquid chromatography detection.

High Performance Liquid Chromatography

DA levels were quantified using a high-performance liquid chromatography system with electrochemical detection (HPLC-ECD; UltiMate 3000 system, Thermo Fisher Scientific, Waltham, MA). Prepared separation was achieved using a mobile phase

consisting of a phosphate buffer containing 0.05 mM EDTA, 1.7 mM orthosilicic acid (OSA), 90.0 mM Na2HPO4, and 50.0 mM citric acid, at a flow rate of 0.2 ml/min. The ECD was equipped with an ANTEC DECRARD SDC detection cell set at 1700 mV, with the guard cell set at 1750 mV. Data acquisition and analysis were performed using the Chromeleon chromatography workstation software (Thermo Fisher Scientific). Peaks and relative concentrations were determined by comparing them to known external standards.

RNA extraction and sequencing

RNA extraction: Sequencing sample come from four different litter sources for each group. Within each litter, we sequenced two rats: one sampled in the native state and the other sampled after self-administration. Rats were perfused with 50 mL of PBS at 4°C. After the PBS perfusion was completed, the brain was quickly removed. Brains were placed in frozen rat brain molds, the brains were cut into 1 mm slices with razor blades, and the brain nucleus was isolated on ice. The isolated brain nuclei were rapidly placed and homogenized in Trizol (Vazyme) at 4°C. After the homogenization, chloroform was added for centrifugation, the supernatant was taken, isopropanol and linear acrylamide were added, and the mixture stand at -80 °C for 2 h. Centrifuge the mixture and separate the RNA precipitate, wash the RNA precipitated with 75% ethanol, evaporate the alcohol at room temperature after centrifugation again, dissolve the RNA precipitate with RNase-free water after the precipitate is dry, and store it at -20°C.

Reverse transcription and quantitative real-time PCR: Reaction mix containing 500 ng RNA, gDNA wiper, oligodT_{23}VN primer and random hexamer were first subjected

to genomic DNA decontamination at 42 ℃ for 2 min, and were then subjected to reverse transcription using Hiscript II reverse transcriptase at 50 °C for 15 min, and then at 85 °C for 2 min according to manufacturers' instructions (R212, Vazyme). For qPCR experiments, 0.2 ul of cDNA product was used. The PCR condition follows 95 °C 30s, (95 °C 10s, 60 °C 30s) × 40cycles in BioRad CFX Opus 384 thermocycler.

Target Gene	Sequence	
Pou3f4	F-	GTGTCAAGGGCGTACTGGAA
	R-	GCGAATAAACCTCGTGTGGC
Rgs9	F-	GGCCAAAGACTTTTGGAATGGA
	R-	CCATCTGGTTGGCAAGGAGT
Myt1l	F-	GGACCAGTCTCCAAGACAGC
	R-	AGTATGGCTTTTTGACATGGCT
Grm5	F-	GAAACCCTAAGCTCCAACGGA
	R-	TGATGTGGACAGACAGTCGC
Neurod6	F-	TCTGCTCACGTTCGTCCAAA
	R-	CTCCTGGCGTTGAGCTGTAA
Igf1r1	F-	TGATCATTGCTCTGCCGGTT
	R-	CACTCTGGTTTCAGGCTCGT

Library preparation and sequencing: The concentration and quality of purified total RNA were determined on Qubit 3.0 and by Agilent 2100 Bioanalyzer, respectively. Total RNA libraries were performed using Ribo-off rRNA Depletion Kit

(Human/Mouse/Rat) (Vazyme #N406) and VAHTS Universal V8 RNA- seq Library Prep Kit for Illumina (Vazyme #NR605), according to the manufacturer's instructions. Briefly, 100 ng total RNA was incubated with rRNA

probe to form an rRNA-probe hybrid, which was depleted by RNase H. Then the probe was removed by DNase I digestion. Then the rRNA-depleted RNA was subjected to fragmentation. The general library was prepared by the inclusion of dNTP in cDNA synthesis and ligation along with an RNA Adapter. Size selection was performed to selectively enrich the insertion size range from 200 bp to 450 bp. The sequencing was performed using Hiseq 3000 by multiplexing samples with dual barcoding index and the sequencing depth was 50 million reads on average.

Bioinformatics analysis

Data processing: Trimmomatic [1] (V0.32) was used to examine the sequencing quality and trim reads, low quality (Phred score < 30) nucleotides or too short reads (< 50 bp) were filtered. Fastqc was used to check the quality of all sequencing data. If abnormal sequence repeats were present, the sample did not pass quality control. All samples including 42 groups passed quality control (QC) except 10 samples (1 naïve, CSA-F1, dStr; 1 naïve, CSA-F1, dHip; 1 naïve, CY-F1, OFC; 1 naïve, CY-F1, mPFC; 1 naïve, CY-F1, NAc; 1 naïve, CY-F1, VTA; 1 SA, CY-F1, OFC; 1 SA, CY-F1, mPFC; 1 SA, CY-F1, NAc; 1 SA, CY-F1, VTA). Paired-end reads were mapped to the UCSC rn6 annotation using Hisat2 (UCSC rn6) [2] (version 2.0.2). Gene annotation and reads counting were performed using featureCounts [3].

Differential gene expression analysis: For each comparison across groups or states,

we perform variance-stabilizing transformation independently using DESeq2 [4] (version 1.26.0) to normalize count data (VSD). For the comparison of gene expression between groups construction of a gene co-express network [5], count data of all samples in the network were performed variance-stabilizing transformation together. Differential expression genes (DEGs) were filtered using a standardized criterion, with a significance threshold of P-value < 0.05 indicating a significant difference, and a minimum differential expression threshold of 20% (Log2(Fold-Change) > 0.26 or < -0.26), mean count > 5 were used to filter genes with low expression and/or detected only in minority samples. The degree of differential expression between samples was assessed based on the range within or between groups. To ensure that the differentially expressed genes were primarily a result of expression differences between groups rather than within groups, density plots were generated. These plots depicted the within-group and between-group ranges of all DEGs across all seven brain regions, confirming that the observed differential expression was attributed to intergroup variations (Figure S2).

The rank-rank hypergeometric overlap test: The rank-rank hypergeometric overlap [6,7] (RRHO) test was used to evaluate the degree of overlap (p-values distinguishing between positive and negative) in gene signatures across brain regions in different parental drug-seeking experiences controlled by SSA-F1 with pattern genes separately, as well as comparing CSA-F1 and CY-F1 for every brain region.

Gene co-expression network analysis: We employed weighted gene co-expression network analysis [5] (WGCNA) to identify co-expressed gene modules using RNA-seq expression data that were first normalized by variance-stabilizing transformation

independently using DESeq2. For single nuclei gene co-expression network construction, the VST matrix contained 22019 genes, median absolute bias (MAD) was used to eliminate the genes that have no differences across all samples, and the remaining 16,514 genes were used to construct the gene co-expression network. For multi-tissue gene co-expression network construction, 1466 pattern genes were used to construct the gene co-expression network.

We performed Pearson correlations between all gene pairs and converted the correlation matrix into a scale-free unsigned adjacency matrix using a power function. the adjacency matrix was further transformed into a topological overlap matrix. Topological overlap between two genes reflects their interactions through all other genes in the network, this approach helps create more cohesive and biologically more meaningful modules. To identify modules of highly co-regulated genes, we used average linkage hierarchical clustering to group genes based on the topological overlap of their connectivity, followed by a dynamic cut-tree algorithm to dynamically separate clustering dendrogram branches into gene modules. Each module was assigned a unique (and arbitrary) color identifier. Gene with the highest connectivity (K-within > 0.6, K-total > 10) were considered hub genes.

Next, we calculate enrichments of pattern genes in the module to estimate the influence of parental drug-seeking experience on modules. Modular differential connectivity across gene co-expression networks was calculated using DGCA [8]. Gene pair is recorded as differential connected if the correlation of the gene pair is significantly different (P-value < 0.01). The ratios of counts of differential connectivity

gene pairs to modular size were used to assess modular differential connectivity (MDC) across gene co-expression networks.

Gene ontology enrichment and transcription factor prediction: Enrichment of gene ontology (GO) terms was performed using the R package cluster profile [9]. Transcription Factor enrichment analysis list transcription factors based on previously annotated transaction factor targets assembled from multiple resources within chEA3 [10].

Statistical analysis

We use R 3.6.2, Stata 16, and GraphPad Prism 9 for statistical analysis and the experimental data are expressed as mean ± standard error (SEM). self-administration in the FR phase was all analyzed by a mixed linear repeated measures model (MMRM), and the comparison between multiple groups was analyzed by one-way analysis of variance (ANOVA). Univariate analysis comparisons between the two groups were performed using a Wilcoxon rank-sum test. All statistical results were defined as statistically significant at $P < 0.05$.

Code availability:

All other data supporting the findings of this study are available within the manuscript and its supplementary files or are available from the corresponding author on request

References

1. Bolger, A. M., Lohse, M. & Usadel, B. Trimmomatic: a flexible trimmer for Illumina sequence data. *Bioinformatics* **30**, 2114-2120, doi:10.1093/bioinformatics/btu170 (2014).
2. Kim, D., Paggi, J. M., Park, C., Bennett, C. & Salzberg, S. L. Graph-based genome alignment and genotyping with HISAT2 and HISAT-genotype. *Nature Biotechnology* **37**, 907-+, doi:10.1038/s41587-019-0201-4 (2019).
3. Liao, Y., Smyth, G. K. & Shi, W. featureCounts: an efficient general purpose program for assigning sequence reads to genomic features. *Bioinformatics* **30**, 923-930, doi:10.1093/bioinformatics/btt656 (2014).
4. Love, M. I., Huber, W. & Anders, S. Moderated estimation of fold change and dispersion for RNA-seq data with DESeq2. *Genome Biol* **15**, 550, doi:10.1186/s13059-014-0550-8 (2014).
5. Zhang, B. & Horvath, S. A general framework for weighted gene co-expression network analysis. *Stat Appl Genet Mol Biol* **4**, Article17, doi:10.2202/1544-6115.1128 (2005).
6. Plaisier, S. B., Taschereau, R., Wong, J. A. & Graeber, T. G. Rank-rank hypergeometric overlap: identification of statistically significant overlap between gene-expression signatures. *Nucleic Acids Research* **38**, doi:10.1093/nar/gkq636 (2010).
7. Cahill, K. M., Huo, Z., Tseng, G. C., Logan, R. W. & Seney, M. L. Improved identification of concordant and discordant gene expression signatures using an updated rank-rank hypergeometric overlap approach. *Sci Rep* **8**, 9588, doi:10.1038/s41598-018-27903-2 (2018).
8. McKenzie, A. T., Katsyv, I., Song, W. M., Wang, M. H. & Zhang, B. DGCA: A comprehensive R package for Differential Gene Correlation Analysis. *BMC Systems Biology* **10**, doi:10.1186/s12918-016-0349-1 (2016).
9. Yu, G. C., Wang, L. G., Han, Y. Y. & He, Q. Y. clusterProfiler: an R Package for Comparing Biological Themes Among Gene Clusters. *Omics-a Journal of Integrative Biology* **16**, 284-287, doi:10.1089/omi.2011.0118 (2012).
10. Keenan, A. B. *et al.* ChEA3: transcription factor enrichment analysis by orthogonal omics integration. *Nucleic Acids Research* **47**, W212-W224, doi:10.1093/nar/gkz446 (2019).

Supplementary figures

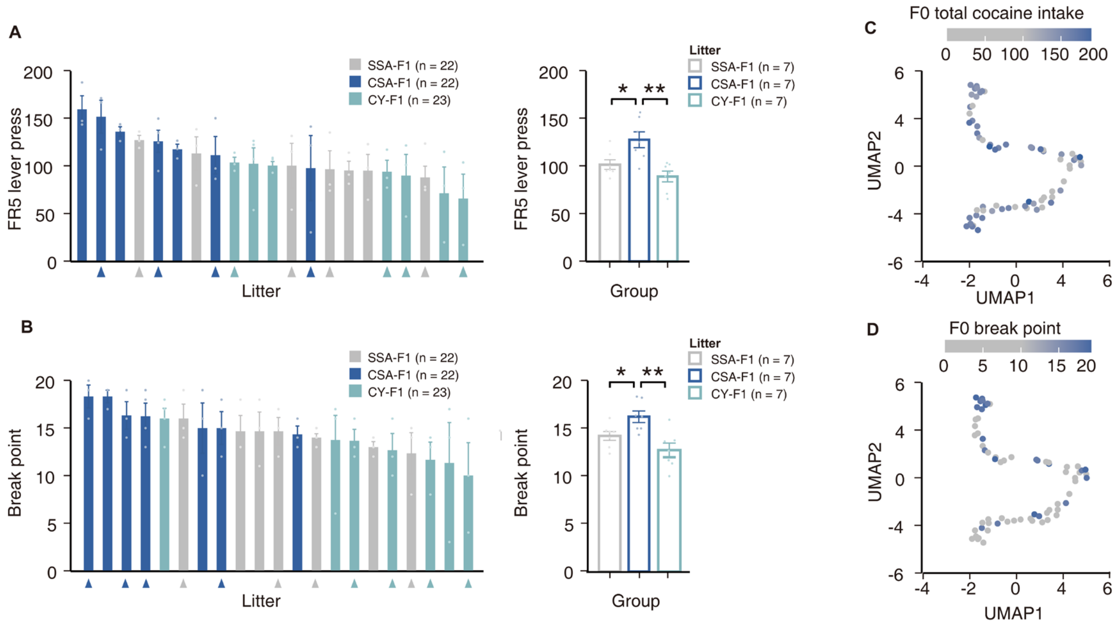

Figure S1. Behavioral performance of F1 rats in each litter.

(A) Self-administration performance of the F1 generation across different litters. Each bar represents a specific litter (left), and the hollow columns (right) summarize the average lever press in FR5 session. (left) each point represents an independent individual, individuals of different litter were statistics respectively. The litters used for sequencing and biological validation in this paper were marked. SSA-F1, n = 22; CSA-F1, n = 22, CY-F1, n = 23. (right) Analysis of group differences in average lever press according to litters, $P_{CSA\text{-}F1\ vs\ CY\text{-}F1} = 0.007$, $P_{CSA\text{-}F1\ vs\ SSA\text{-}F1} = 0.029$, Wilcoxon rank-sum test.

(B) Each bar represents a specific litter (left), and the hollow columns (right) summarize the average lever press in PR session. (left) each point represents an independent individual, individuals of different litter were statistics respectively. The litters used for

sequencing and biological validation in this paper were marked. SSA-F1, n = 22; CSA-F1, n = 22, CY-F1, n = 23. (right) Analysis of group differences in break point according to litters, $P_{CSA\text{-}F1\ vs\ CY\text{-}F1} = 0.007$, $P_{CSA\text{-}F1\ vs\ SSA\text{-}F1} = 0.021$, Wilcoxon rank-sum test.

(C, D) Clustering of F1 individuals by UMAP based on the self-administration performance. The color represents the (C) paternal break point and the (D) total drug intake.

*$P < 0.05$, **$P < 0.01$. Data are shown as mean ± s.e.m.

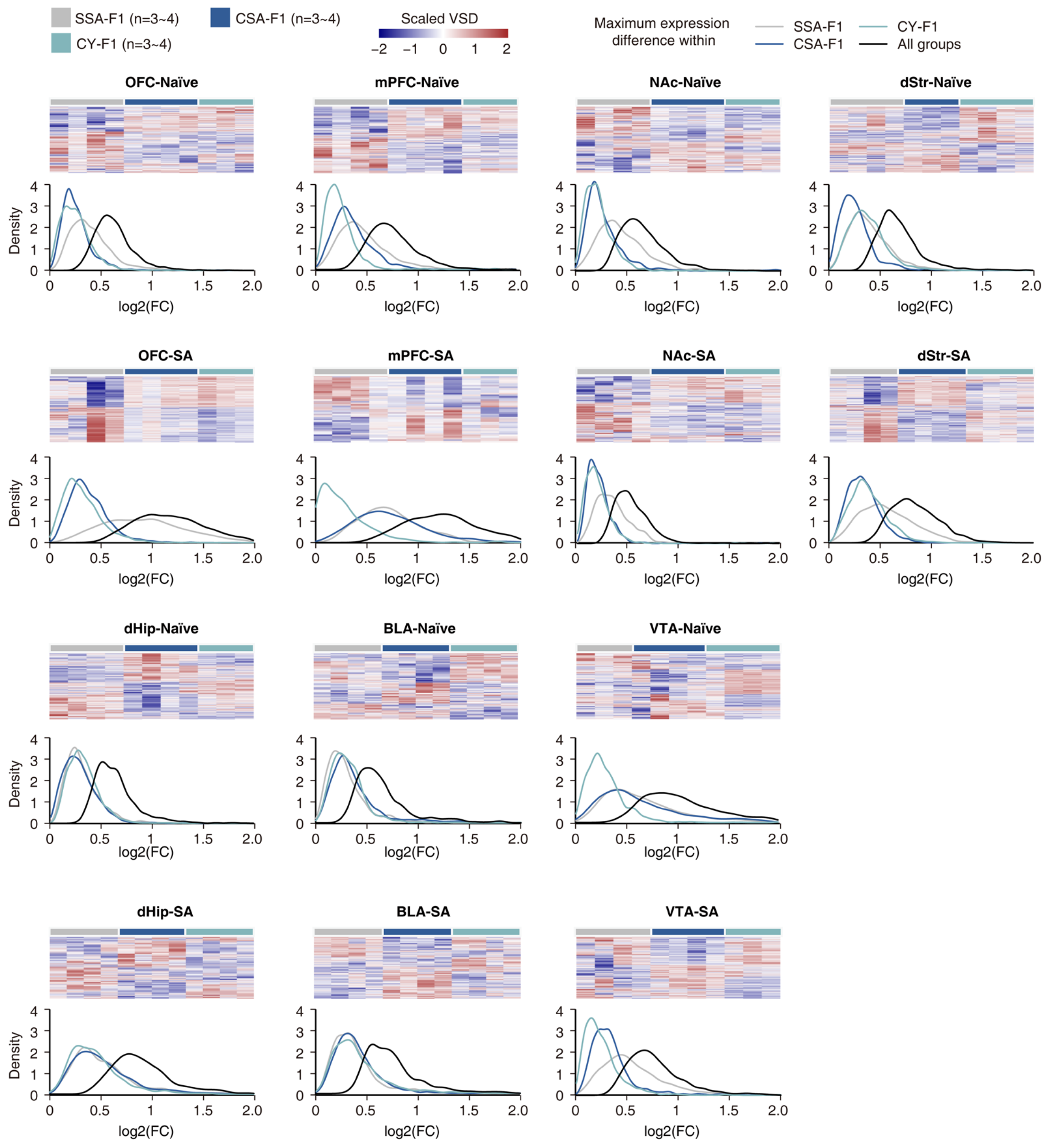

Figure S2. Overview of differentially expressed genes in each sample of each brain region

Heatmaps displays the expression profiles of DEGs in all seven brain regions. Each panel represents a specific brain region, and within each panel, the samples from different groups are represented by individual columns (grey = SSA-F1, blue = CSA-F1, light blue = CY-F1), each row represents a DEG, density plots show the range of

expression values for DEGs within each group (grey = SSA-F1, blue = CSA-F1, light blue = CY-F1) and between groups (black).

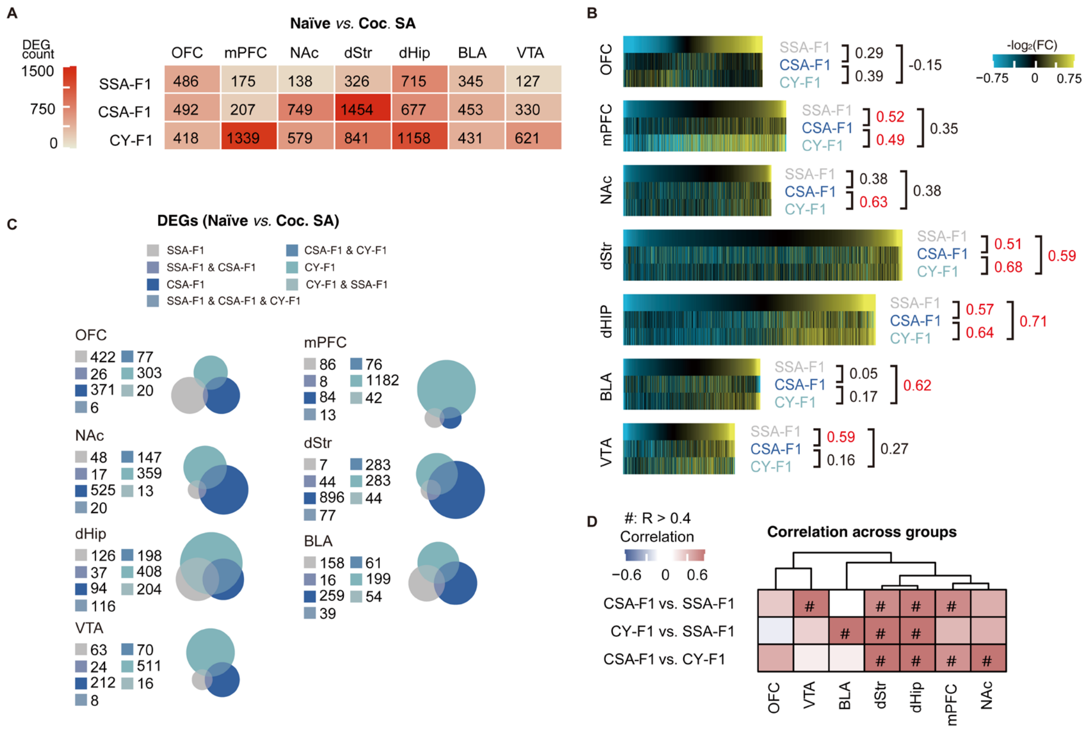

Figure S3. Transcriptional responses to cocaine self-administration are disrupted by paternal cocaine-seeking experience in the reward circuitry.

(A) Differential expression analysis was performed to identify differentially expressed genes (DEGs) across cocaine self-administration (Coc. SA vs Naïve) in each group individually. Differentially expressed genes with at least a nominal P ≤ 0.05. Several differentially expressed genes per brain region are indicated (red = greatest, gray = least).

(B) Heatmaps show DEGs in response to cocaine self-administration for reward circuitry (yellow = upregulation; blue = downregulation). The correlation of $\log_2$(FC) between groups is marked on the right. Positive correlation (R > 0.4) were marked as red.

(C) Venn diagrams of differentially expressed genes in SSA-F1, CSA-F1, and CY-F1 after cocaine self-administration for the reward circuitry.

(D) Correlation analysis between groups using $\log_2$(FC) of differential genes across states (Coc. SA vs. Naïve) in each brain region (#R > 0.4).

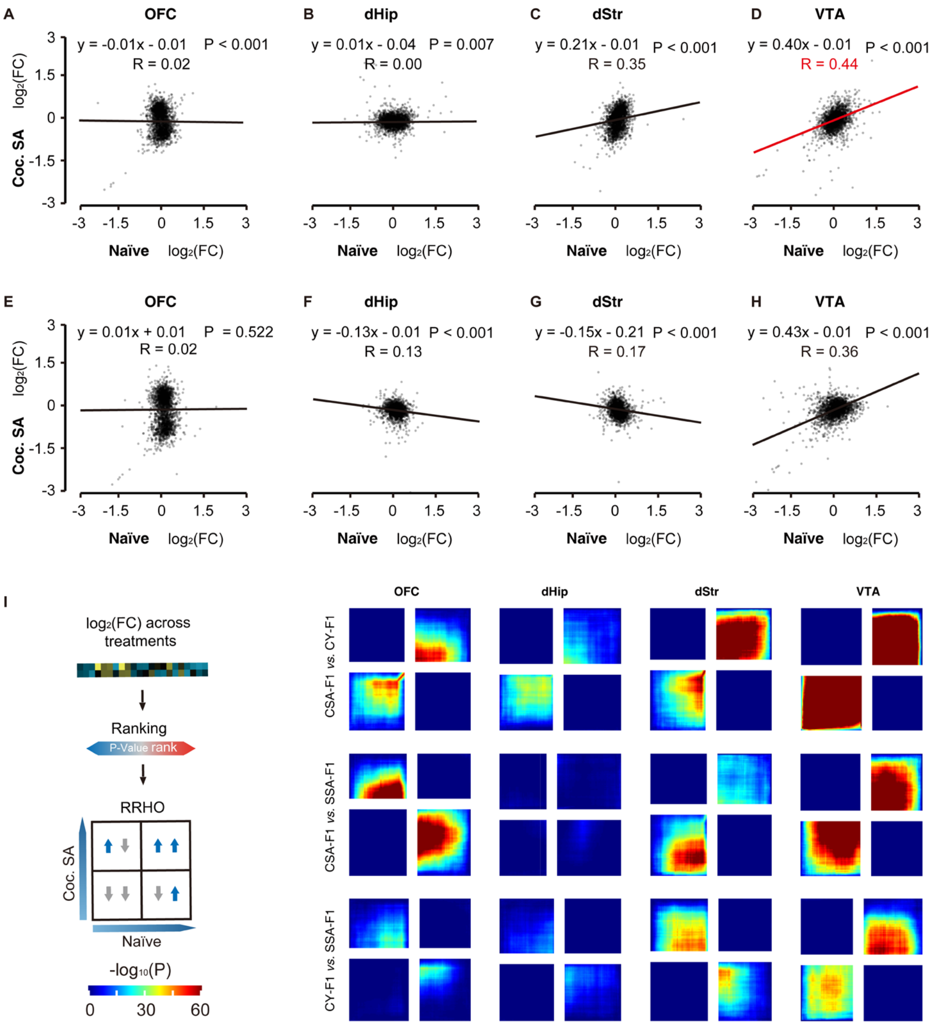

Figure S4. Transcriptional stability between naïve and Coc. SA states in OFC, dHip, dStr, and VTA.

(A-D) Correlation of log2(FC) (CSA-F1 vs. SSA-F1) between naïve and Coc. SA states in OFC (A), dHip (B), dStr (C), and VTA (D).

(E-H) Correlation of log2(FC) (CY-F1 vs. SSA-F1) between naïve and Coc. SA states in OFC (E), dHip (F), dStr (G), and VTA (H).

(I) RRHO analysis. Then RRHO across states (Naïve vs. Coc. SA) was plotted based on the rank of (+/-) P-value in each brain region. The color represents the degree of

significance of the overlap between two brain regions.

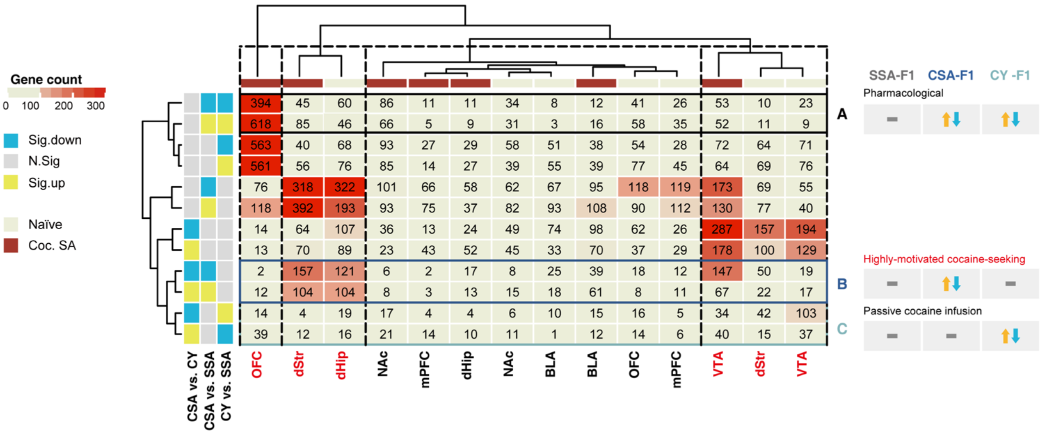

Figure S5. Summary of pattern analysis.

Table of counts of all 12 expression patterns in 7 reward-associated brain regions. Expression patterns were listed in the left frame (blue = down-regulation, yellow = up-regulation). The count of each expression pattern is listed (red = greatest, gray = least). Three expression patterns with clear significance were screened out.

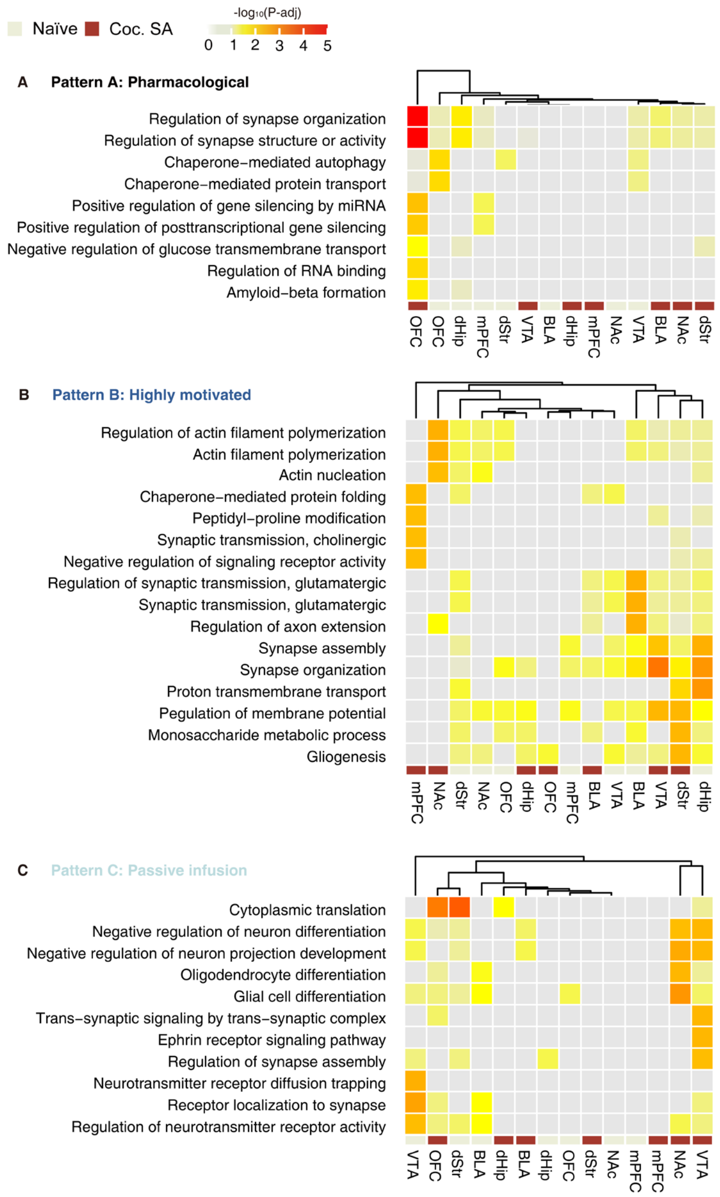

Figure S6. GO enrichment of pattern genes in all brain regions

The heatmap showed the enrichment of pattern genes in all seven brain regions. The color represents the degree of enrichment for pattern genes, the light green notes indicate naïve state, dark green indicate Coc.SA state

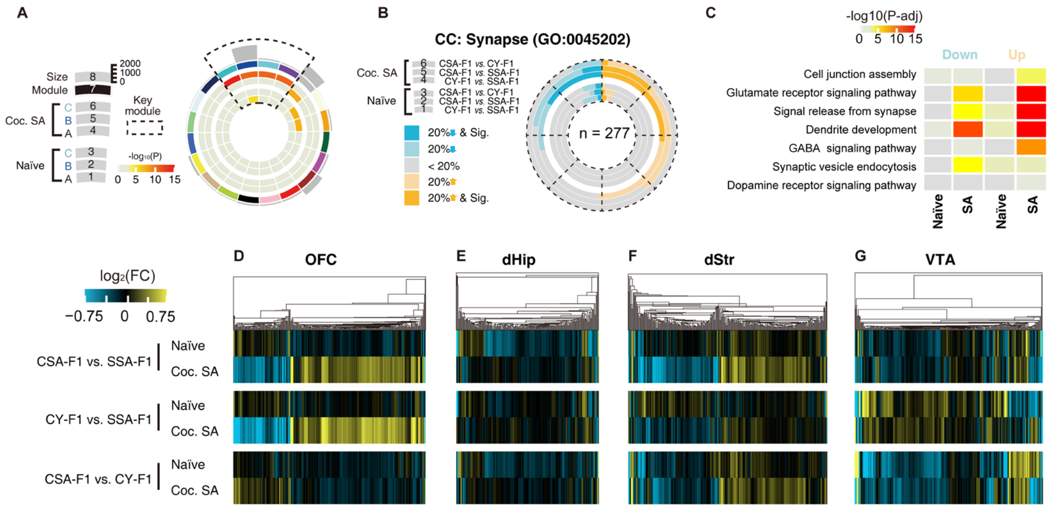

Figure S7. Co-expression analysis of OFC and the landscape of transcriptional difference across groups

(A) Circos plots for the WGCNA result of OFC. Each slice of the chart represents a gene co-expression module. The key for the concentric circles is shown in the middle of the figure. The outermost represents the modular size of the slice. The secondary outer rectangle is an arbitrary color as the module name, The inner concentric circles represent the degree of enrichment for pattern genes (colors reflect FET P-values). Modules selected for subsequent analysis are marked with dashed lines.

(B) Pie charts of group differences of OFC in synaptic-enriched genes within the screened module before and after cocaine self-administration (blue = significantly down (20% downregulate, $P < 0.05$), light blue = Not significantly down (20% downregulate, $P > 0.05$), yellow = significantly up (20% upregulate, $P < 0.05$), light yellow = Not significantly up (20% upregulate, $P > 0.05$).

(C) Enrichment analysis for the representative subset of synaptic function in OFC

(colors reflect adjusted P-values).

(D-G) Heatmaps show the expression of genes in the GO term “synapse” in (D) OFC, (E) dHip, (F) dStr, and (G) VTA (yellow = upregulated; blue = downregulated).

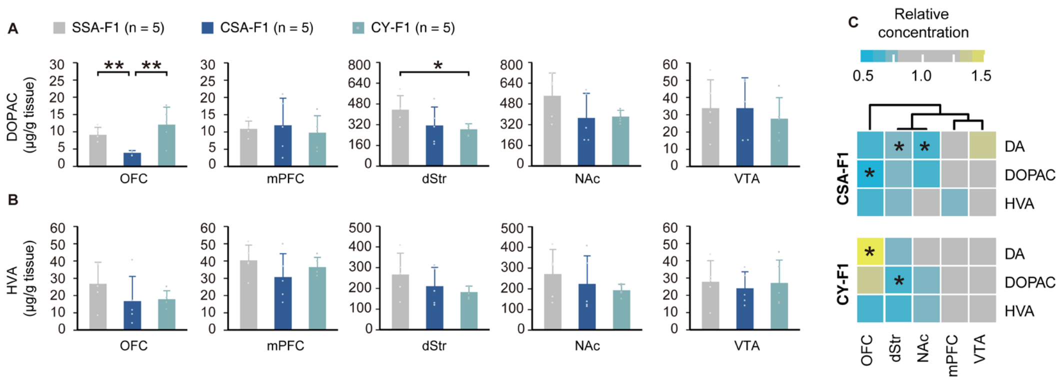

Figure S8. The concentration of dopamine metabolites in F1 generation.

(A) Concentration of dihydroxy-phenyl acetic acid (DOPAC) in VTA and its major downstream of naïve F1 rats, *P < 0.05, **P < 0.01, SSA-F1 n=5; CSA-F1 n=5; CY-F1 n=5.

(B) Concentration of herpes virus ateles (HVA) in VTA and its major downstream of naïve F1 rats, SSA-F1 n=5; CSA-F1 n=5; CY-F1 n=5.

(C) Relative concentration (based on SSA-F1) of dopamine and dopamine matabolites in VTA and its major downstream (right) blue=relative decreased, yellow=relative increased, *P < 0.05, SSA-F1 n=5; CSA-F1 n=5; CY-F1 n=5.

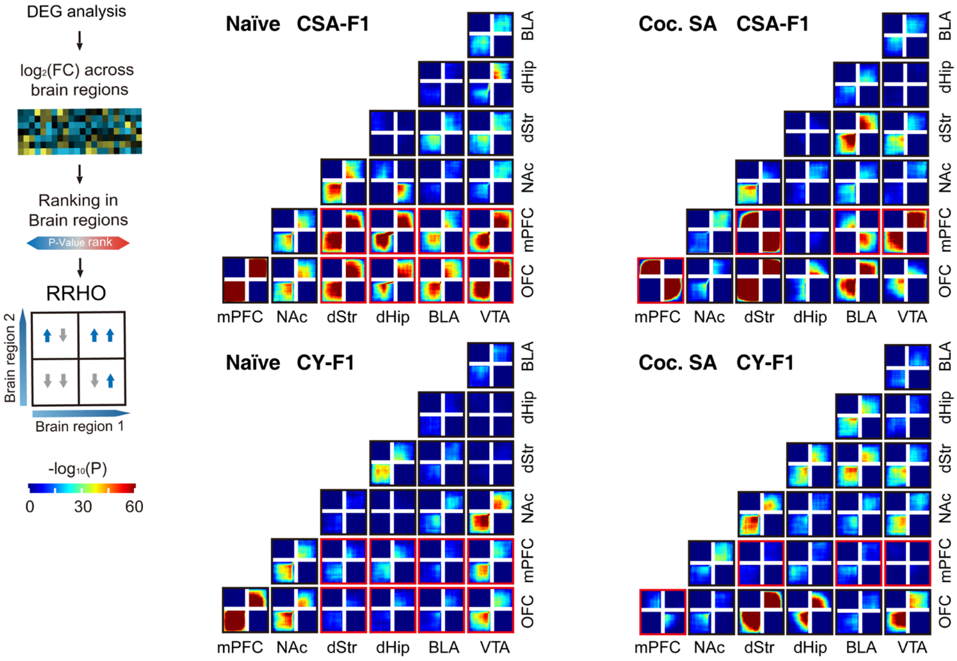

Figure S9. Transcriptional change correlation across brain regions in CSA-F1 revealed by RRHO.

RRHO analysis. Overview of rank-rank hypergeometric overlap (RRHO) across brain regions (left). Amplitudes of expression changes in CSA-F1 and CY-F1 compared to SSA-F1 in each brain region under naïve or Coc.SA conditions. Then RRHO across brain regions was plotted based on the rank of (+/-) P-value in each brain region. The color represents the degree of significance of the overlap between two brain regions (right).

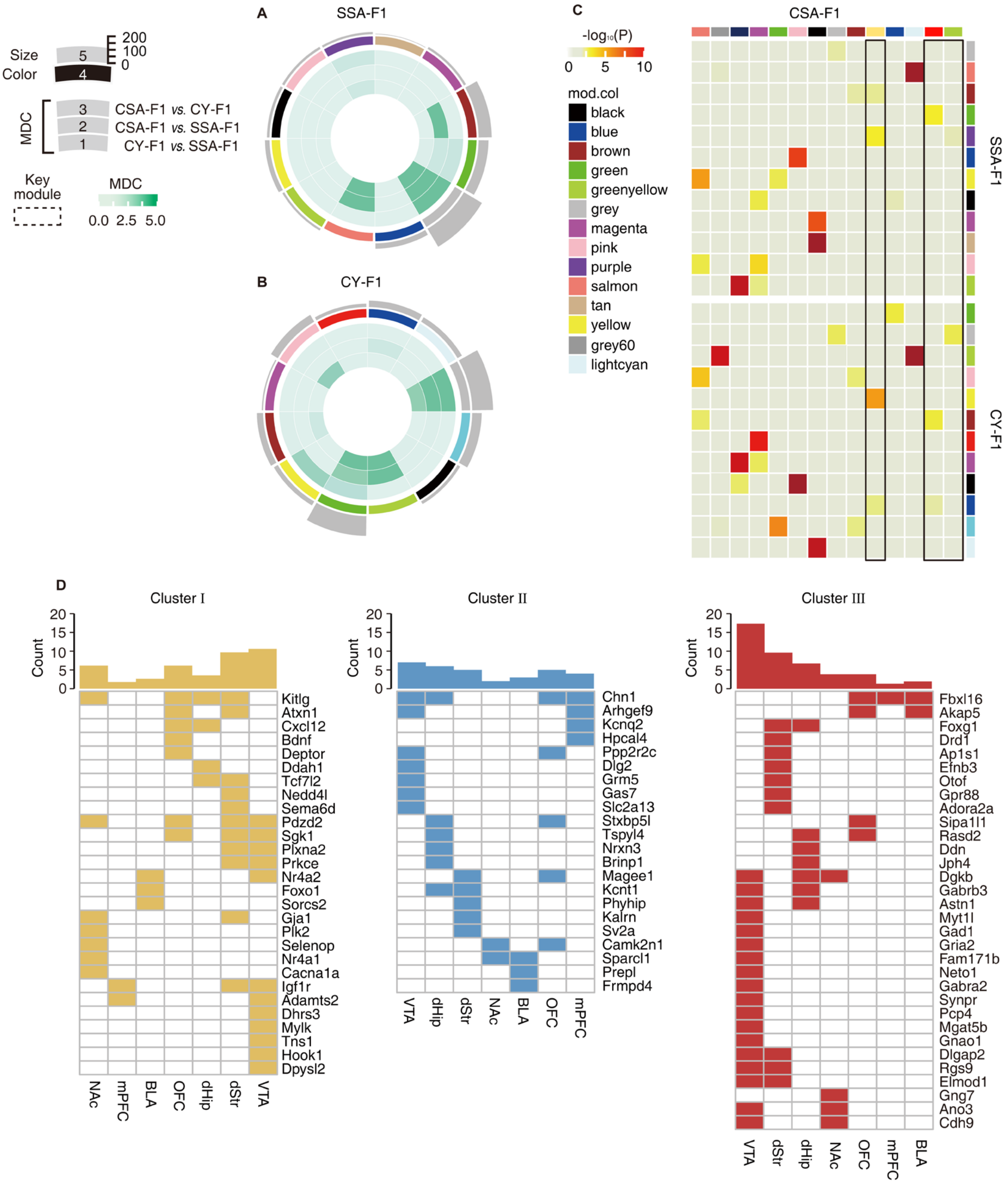

Figure S10. Intergroup network structure analysis and the brain region distribution of clustered genes.

(A-B) Circos plots for the WGCNA result of SSA-F1(a) and CY-F1(b) with seven reward-associated brain regions. The meaning of each component in the figure is the same as that of Figure 6A.

(C) The degree of overlap between gene co-expression network modules in SSA-F1, CSA-F1, and CY-F1 (colors reflect corrected FET P-values, red = high coincidence, gray = low coincidence). The arbitrary color of each modular was shown on the boundary of the heatmap.

(D) Plots show the brain region distribution of genes in clusters, the color represents the type of clusters, and gene names are exhibited on the right of the heatmaps. The column plots summarize the gene counts in each brain region.

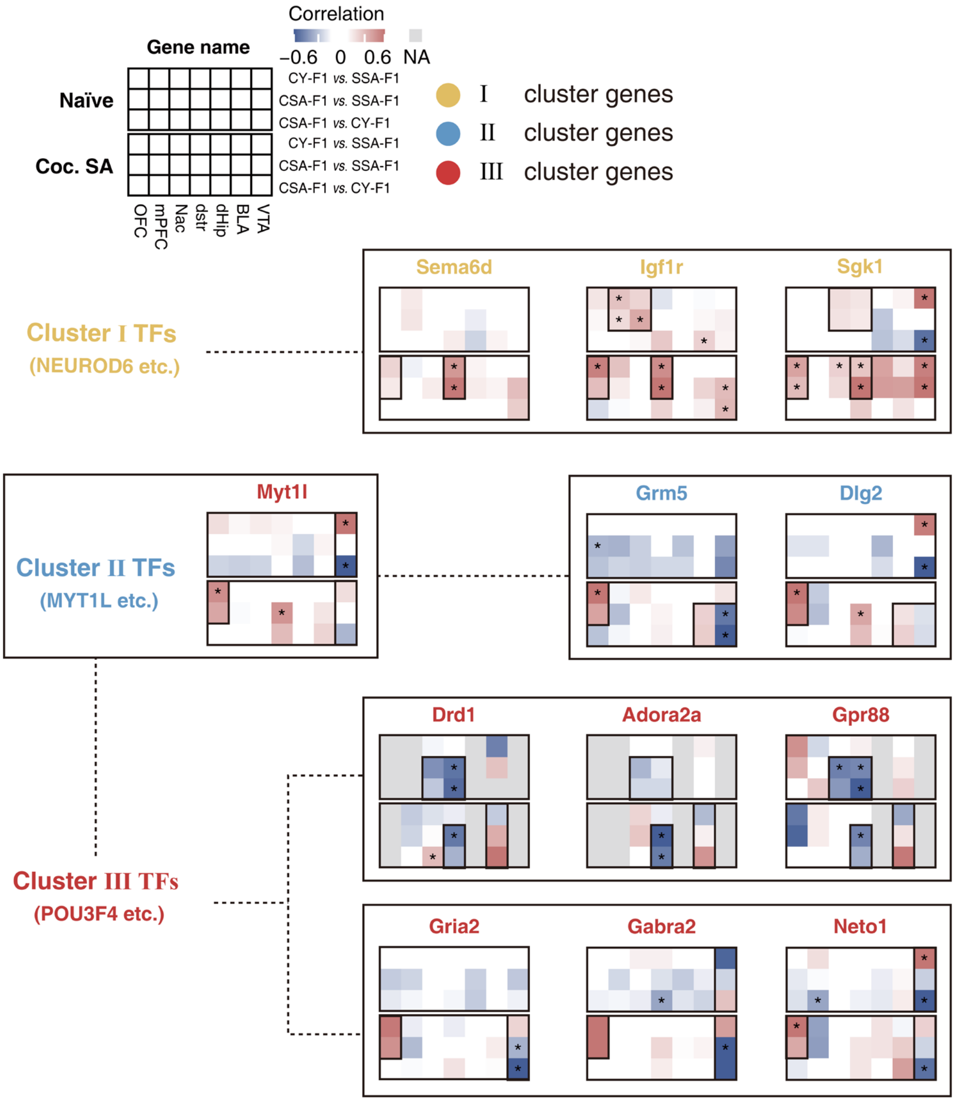

Figure S11. Between-group variation of representative genes at a whole-brain scale.

Visualization of representative genes in screened modules presents that even though single genes have different between-group difference patterns in multi-brain regions, there are similarities of multi-gene expression differences at the whole-brain scale. The potential transcription cascade was also presented. Genes under different regulation clusters or sever different functions show distinct transcriptional patterns on the whole-brain.

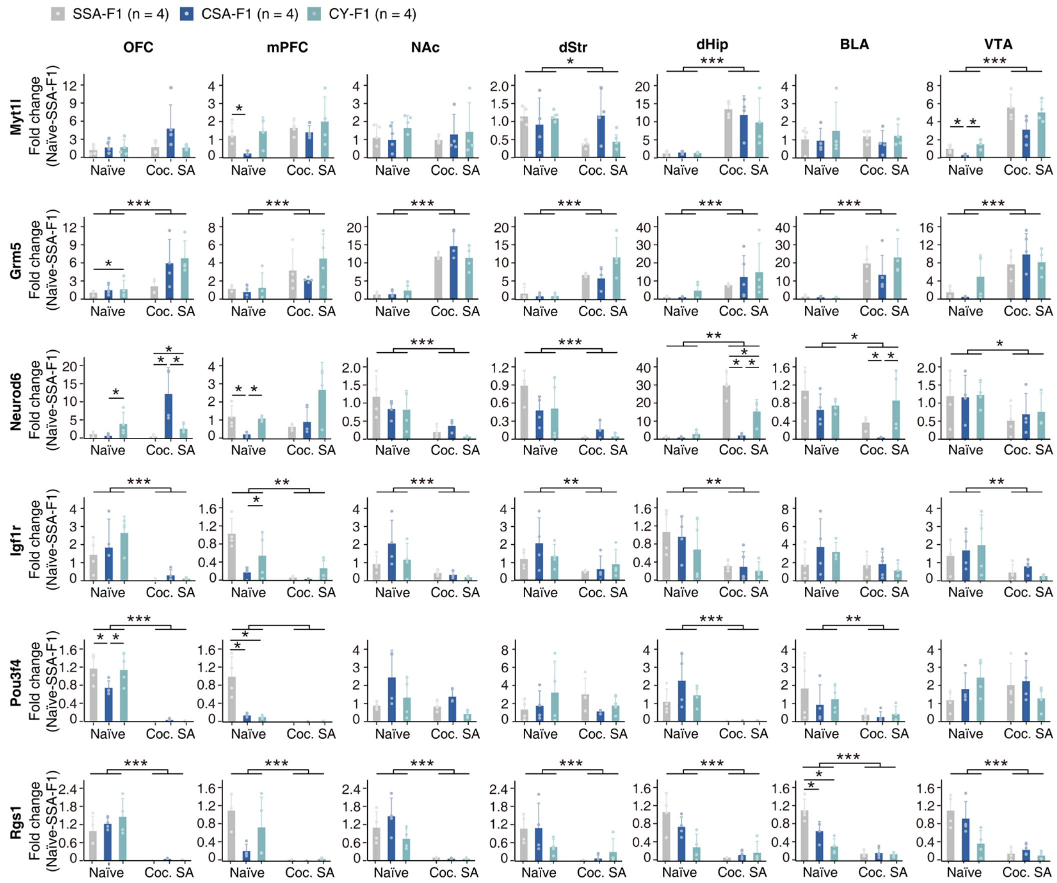

Figure S12. qPCR validation of candidate genes.

We did independent qPCR validation of the candidate genes selected by gene co-expression network analysis, and each group contained four biological replicates. For each gene, we calculated the relative expression using the expression of SSA-F1 in the Naïve state as a metric. SSA-F1, n = 4; CSA-F1, n = 4, CY-F1, n = 4, Wilcoxon rank-sum test.

*P < 0.05, **P < 0.01, ***P < 0.001. Data are shown as mean ± s.d..

Module	Go term	P.adjust	Gene ratio
Cyan	Negative regulation of microtubule polymerization	1.53E-03	6/428
	Regulation of proteolysis involved in cellular protein catabolic process	1.53E-03	19/428
	Negative regulation of microtubule polymerization or depolymerization	1.53E-03	9/428
Blue	Synapse organization	2.83E-19	181/3446
	Dendrite development	8.09E-15	123/3446
	Positive regulation of cell projection organization	2.66E-14	170/3446
Light cyan	Regulation of fibrinolysis	0.643	3/229
	Mating	0.643	5/229
	Response to pain	0.643	4/229
Purple	Membrane protein proteolysis	0.383	7/373
	Membrane protein ectodomain proteolysis	0.441	6/373
	Regulation of endoplasmic reticulum stress-induced intrinsic apoptotic signaling pathway	0.515	5/373

Table S1. Enrichment of biological pathways in selected modules of OFC

Module	Go term	P.adjust	Gene ratio
Pink	Regulation of cell growth	0.134	18/251
	Negative regulation of neuron differentiation	0.271	12/251
	Negative regulation of cell growth	0.271	10/251
Green	Learning or memory	1.15E-05	22/442
	Cognition	2.14E-05	23/442
	Regulation of GTPase activity	2.59E-05	25/442
Blue	Negative regulation of cell projection organization	2.87E-08	50/1858
	Regulation of cell morphogenesis involved in differentiation	4.98E-08	70/1858
	Synapse organization	9.58E-08	86/1858
Black	Peptidyl-threonine modification	3.66E-03	35/2065
	Regulation of membrane potential	3.66E-03	88/2065
	Peptidyl-threonine phosphorylation	3.66E-03	33/2065
Midnight blue	Inositol phosphate metabolic process	0.051	5/110
	Inositol phosphate dephosphorylation	0.051	3/110
	Cellular carbohydrate metabolic process	0.051	9/110
Light green	Fertilization	0.041	6/63
	Positive regulation of TOR Signaling	0.172	3/63
	Bleb assembly	0.172	2/63

Table S2. Enrichment of biological pathways in selected modules of dStr

Module	Go term	P.adjust	Gene ratio
Tan	Positive regulation of cell projection organization	0.086	14/158
	Ionotropic glutamate receptor signaling pathway	0.086	4/158
	Neuron death	0.086	13/158
Black	Ribosomal large subunit biogenesis	0.809	10/564
	Antibiotic biosynthetic process	0.809	5/564
	Ribonucleoprotein complex biogenesis	0.809	27/564
Purple	Cilium movement	3.91E-03	8/230
	Inorganic cation import across plasma membrane	9.20E-03	8/230
	Inorganic ion import across plasma membrane	9.20E-03	8/230
Blue	Regulation of membrane potential	2.54E-04	125/3059
	Synapse organization	5.07E-04	124/3059
	Synaptic vesicle cycle	3.21E-03	71/3059

Table S3. Enrichment of biological pathways in selected modules of dHip

Module	Go term	P.adjust	Gene ratio
	Synapse organization	6.44E-13	86/1259
Red	Regulation of membrane potential	1.33E-11	82/1259
	Synaptic vesicle cycle	1.95E-09	51/1259
	Protein folding	7.86E-04	19/508
Green yellow	Protein refolding	4.73E-03	8/508
	Positive regulation of cellular protein localization	0.012	24/508
	Vesicle-mediated transport in synapse	1.84E-08	45/960
Blue	Synaptic vesicle cycle	1.33E-07	41/960
	Synapse organization	1.49E-06	59/960
	Extracellular matrix organization	1.80E-21	64/943
Brown	Extracellular structure organization	1.80E-21	69/943
	Wound healing	4.69E-20	81/943

Table S4. Enrichment of biological pathways in selected modules of VTA
